# Supplementary material for: Aneuploidy in Oocytes Is Prevented by Sustained CDK1 Activity through Degron Masking in Cyclin B1
Source: Dev Cell. 2019 Mar 11;48(5):672–684.e5. doi: 10.1016/j.devcel.2019.01.008 (PMC6416240; doi:10.1016/j.devcel.2019.01.008)
Supplement: Document S1. Figures S1–S7 and Table S1 [file mmc1.pdf]

Developmental Cell, Volume *48*

Supplemental Information

Aneuploidy in Oocytes Is Prevented by Sustained CDK1 Activity through Degron Masking in Cyclin B1

Mark D. Levasseur, Christopher Thomas, Owen R. Davies, Jonathan M.G. Higgins, and Suzanne Madgwick

Supplementary Figure and Table Legends

Figure S1. Related to Fig. 1. Representative raw data destruction profiles of D-box-B1-C (cerulean, pink) and Y170A-B1-V (blue) co-expressed in the same oocyte. (A) in an untreated oocyte, and **(B)** in oocytes on addition of cycloheximide as indicated (CHX)**.** Note that the order of D-box-B1 and Y170A-B1 destruction did not depend on continued protein synthesis (which is prevented by cycloheximide), nor on their relative expression levels (**i + ii** comparison). PB1 extrusion time as indicated.

Figure S2. Further destruction profiles of cyclin B1 truncations and mutations, an extension of Fig. 3. (A) NTH sequence detail and nomenclature of venus tagged cyclin B1 mutants. **(B)** Mean destruction profiles of D-box-B1+NTH (n=22), D-box-B1+NTH DIY-A (n=18) and D-box-B1-V (n=34). **(C)** Mean destruction profiles of n190Y170A-B1 (n=36), n190LRQL-A (n=13), and n190DIY-A (n=21). Cyclin B1 mutants included in parts 'C' carry an additional I150T mutation. This residue falls within cyclin B1's nuclear export signal, a sequence thought to be redundant in oocytes which do not have a nucleus. **(D)** Destruction profiles of Y170A-B1 and PMmut-B1 with (Y170A-B1* and PMmut-B1*) and without (Y170A-B1 and PMmut-B1) an I150T point mutation demonstrating that this amino acid switch did not affect either the timing, or the extent of destruction. Error bars ± SEM throughout.

Figure S3. Related to Fig. 3C. Examples of individual cyclin B1 destruction traces. (A) Raw data demonstrating protein expression ranges are similar for PMmut-B1-V and Y170A-B1-V. Given that the cyclin B1 and fluorescent component of each construct are physically linked, we reasoned that by matching fluorescence levels, different cyclin B1 reporters are expressed at similar levels. Y170A-B1-V expressing oocytes and PMmut-B1-V expressing oocytes are aligned to PB1 extrusion within treatment groups. Mean PB1 extrusion timings between treatment groups are identical. Thin traces represent individual oocytes while heavy traces indicate the mean. **(B)** Raw data above processed to normalize fluorescence values to 100%, 2.5 hours ahead of PB1 extrusion. This time point was selected as the initiation of cyclin B1 destruction in the average oocyte. Note that normalising data does not affect the observed order of destruction. Thin traces represent individual oocytes, while heavy traces indicate the mean. **(C)** Mean destruction profiles of Y170A-B1-V (n=18) and PMmut-B1-V (n=18) after addition of cycloheximide (CHX) at the time indicated. Individual traces were aligned to GVBD and normalised to 100 a.u. at the point of CHX addition. Error bars ± SEM. **(D)** Oocytes 5.5hrs post GVBD expressing Y170A-B1-V and PMmut-B1-V, incubated in the DNA dye SiR-DNA.

Figure S4. Related to Fig. 4. Quantification of cyclin B1 and CDK1 in mouse oocytes 5.5 h post GVBD using a 1:1 cyclin B1:CDK1 protein complex. (A) Western blot of known amounts of cyclin B1 and CDK1 recombinant protein (purified complex) alongside known numbers of mitotic U2OS cells to quantify cyclin B1 and CDK1 protein bands in U2OS cells (cell numbers indicated above). **(B)** Band densities of 'purified complex' lanes in part 'A' plotted relative to protein amount in ng. **(C)** Calculated amounts of cyclin B1 and CDK1 in 1000 U2OS cells from 'A' using the equation from 'B'. **(D)** Western blot of mitotic mouse embryonic fibroblasts (MEFs), U2OS cells, oocytes collected 5.5 hours post GVBD and 1.5 ng of cyclin B1 + 1.5 ng of CDK1 recombinant protein (cell numbers indicated above). Note the difference in the balance of cyclin B1 and CDK1 in the mitotic cell cycles of MEF's where CDK1 is in excess. **(E)** Using the same strategy as in 'B' and 'C', cyclin B1 and CDK1

band densities from part ‘D’ were used to relate cyclin B1 and CDK1 protein levels in oocytes to an equivalent number of U2OS cells. From this cyclin B1 and CDK1 protein amounts were calculated per oocyte. We calculate the ratio of cyclin B1:CDK1 to be approximately 6:1. All mitotic cell lysates are prepared from cells arrested in nocodazole and collected by shake off.

(F) Western blots of control and cyclin B1 morpholino (MO) injected oocytes collected 5.5 hours post GVBD (numbers of oocytes loaded per lane are indicated). The upper blot was probed for cyclin B1, and the lower for CDK1 (2 different exposures). Quantification of protein bands indicates a 67% knockdown of cyclin B1 in cyclin B1 morpholino injected oocytes. The loss of cyclin B1 also results in a 12% loss of CDK1 protein. The ratio of cyclin B1:CDK1 was therefore calculated to be ~2.25:1 in cyclin B1 MO oocytes 5.5 hours post GVBD. Note that CDK1 levels are unchanged between GV stage and 5.5 hours post GVBD. **(G)** Western blot of 40 oocytes injected with Y170A-B1-V, probed for cyclin B1. Oocytes were collected and lysed 5 hours post GVBD. In order to be sure of detecting exogenous cyclin B1, 4-fold greater needle concentrations of cRNA were used for microinjection of blotting oocytes than those used to report cyclin B1 destruction profiles. We determined that all cyclin B1 reporters are expressed at between 8-13% of endogenous at their peak prior to destruction. All western blot images presented are scanned X-ray film.

Figure S5. Related to Fig. 4B. Validation of the CDK1 activity FRET sensor in oocytes. FRET ratios from oocytes expressing a CDK1 activity biosensor are able to detect changes in the balance between cyclin B1:CDK1 kinase activity and antagonistic phosphatase(s) activity. **(A)** Mean FRET ratios in oocytes after: **green trace,** no further treatment (n=72); **blue trace,** addition of the CDK1 inhibitor flavopiridol, demonstrating a rapid reduction in FRET signal (n=12); **yellow trace,** following overexpression of non-destructible cyclin B1 (Δ90 cyclin B1 lacks a D-box, n=23); **red trace,** inactive control biosensor in which the phosphorylated Ser126 is mutated to Ala (n=12). Inhibitors were added to the medium and PBs were extruded at the times indicated.

The Δ90 cyclin B1 experiment demonstrates that the pre-anaphase loss of FRET signal in control oocytes is the result of CDK1-bound cyclin B1 destruction. Oocytes overexpressing Δ90 cyclin B1 show no loss of FRET, indicating a stable balance between CDK1 kinase activity and phosphatase activity. In control oocytes, the loss of CDK1 activity switches the balance in favour phosphatase activity and the FRET ratio drops. This experiment also demonstrates that CDK1 levels, not cyclin B1 levels, are rate limiting since CDK1 activity in oocytes overexpressing Δ90 cyclin B1 does not increase beyond that of control cells.

(B) Mean FRET ratios from a pool of oocytes expressing the CDK1 biosensor following either no further treatment (**green trace** n=18) or microinjection of 2pg of unlabelled recombinant CDK1 protein, (**black trace** n=17/29). Oocytes injected with CDK1 are delayed in PB1 extrusion as indicated, presumably due to the need to disassemble greater numbers of cyclin B1:CDK1 complexes. 12/29 CDK1 protein injected oocytes failed to extrude a polar body and are not included. Given that the loss of CDK1 activity must be abrupt to generate an irreversible anaphase switch (Holt et al., 2008), we reasoned that the time lag in destroying more CDK1 associated cyclin B1 perturbed this switch resulting in a stalled anaphase. This experiment serves to demonstrate that we are working within the dynamic range of the FRET sensor and provides further evidence that CDK1 is rate limiting. Means ± SEM are shown.

Figure S6. Related to Fig 5. D-box substrates are targeted earlier where the amount of excess cyclin B1 is restricted. (A) Example replicate showing mean, raw data, CDK1 activity FRET ratios in untreated (n=18) and cyclin B1 MO injected oocytes (n=26) demonstrating that absolute CDK1 activity does not differ until approximately 2-2.5 hours ahead of polar body extrusion. Time 0 = PB1 extrusion in control oocytes.

Destruction of D-box-B1-V in untreated (n=20) and cyclin B1 MO treated oocytes (n=31) as; **(B)** raw data traces aligned at GVBD, thin traces represent individual oocytes, while heavy traces represent the average; **(C)** GVBD aligned data normalised to 100% at the maximum fluorescence prior to PB1 extrusion. **(D)** Mean destruction profiles of securin-V in control oocytes (n=15 oocytes) and cyclin B1 MO oocytes (n=15 oocytes). Error bars ± SEM.

Figure S7. Related to Fig. 6. Spindle parameter measurements in control and cyclin B1 MO oocytes. Box plots of relative spindle length and width in control oocytes (green) and cyclin B1 MO oocytes (purple) at **(A)** 5.5hrs post GVBD and **(B)** 'pre-anaphase' (the last image collected before anaphase). **(C)** Box plots showing relative chromosome dispersion in control (green) and cyclin B1 MO oocytes (purple) at 5.5hrs post GVBD and 'pre-anaphase'. **(D)** Box plots showing the relative distance of the spindle from the cortex in control (green) and cyclin B1 MO oocytes (purple) at 5.5hrs post GVBD and 'pre-anaphase'. * denotes a significant difference in means P<0.05. ** denotes a significant difference in means P<0.005. **(E)** Given that cyclin B1 MO treated oocytes extrude PBs earlier than control oocytes (~40 minutes earlier), a pre-anaphase measurement (taken a maximum of 15 minutes ahead of anaphase) in cyclin B1 MO oocytes could be argued to be more closely analogous to images of control oocytes taken 30-45 minutes earlier (45-60 minutes' pre-anaphase). Therefore, we quantified changes in chromosome dispersion at earlier time points in control oocytes. We suggest that chromosomes do not simply condense on the metaphase plate within the final 30-45 minutes. Instead, as previously reported (Kitajima et al., 2011), this is a gradual process. We suggest that in cyclin B1 MO oocytes the lack of chromosome congression at anaphase is not simply due to premature cytokinesis.

Table S1. Related to Figs. 5 and 6. P-values summarising the significance of differences in measurements of spindle morphology between time points. Between 5.5 hours post GVBD and pre- anaphase, differences in spindle length and width were not judged to change in either control or cyclin B1 MO oocytes. Between the same time points, chromosomes congressed in control oocytes (P=0.003, green). However, a much more modest reduction in the dispersion of chromosomes was not judged to be significant in cyclin B1 MO oocytes (P=0.097, pink). In both control and cyclin B1 MO oocytes the spindle moved significantly closer to the cortex of the oocyte.

Figure S1

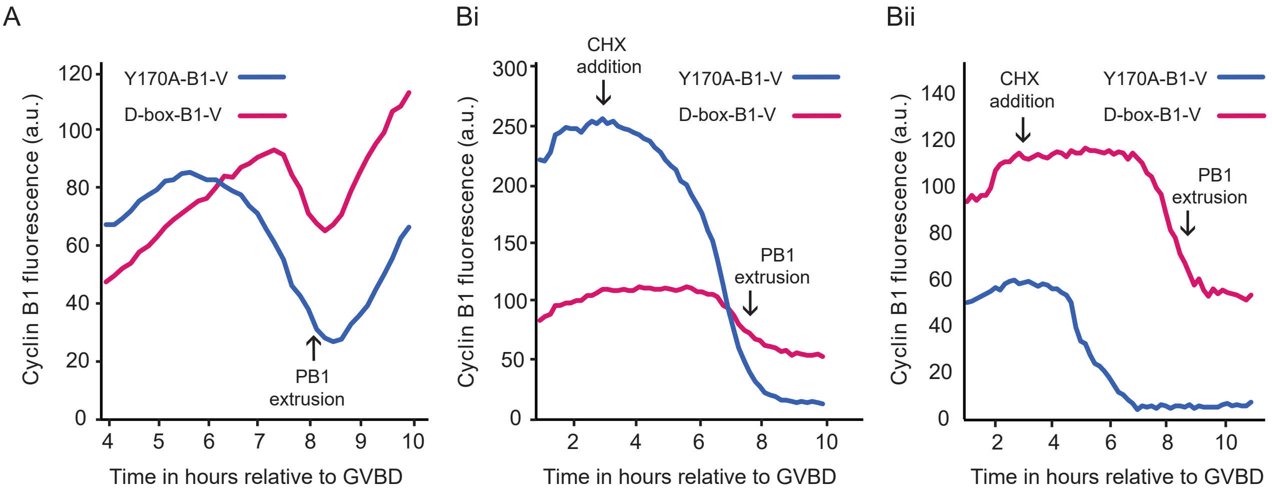
A
Y170A-B1-V
D-box-B1-V
Cyclin B1 fluorescence (a.u.)
0
20
40
60
80
100
120
4
5
6
7
8
9
10
PB1
extrusion
Time in hours relative to GVBD
Bi
CHX
addition
Y170A-B1-V
D-box-B1-V
Cyclin B1 fluorescence (a.u.)
0
50
100
150
200
250
300
2
4
6
8
10
PB1
extrusion
Time in hours relative to GVBD
Bii
CHX
addition
Y170A-B1-V
D-box-B1-V
PB1
extrusion
Cyclin B1 fluorescence (a.u.)
0
20
40
60
80
100
120
140
2
4
6
8
10
Time in hours relative to GVBD

Figure S2

A

Expanded NTH detail

WT-B1	165	N L C S E Y V K D I Y A Y L R Q L E E E Q A V R P K	190
Y170A-B1	165	N L C S E A V K D I Y A Y L R Q L E E E Q A V R P K	190
PMmut-B1	165	N L C S E A V K A A A A Y A A A A E E E Q A V R P K	190
LRQL-A	165	N L C S E A V K D I Y A Y A A A A E E E Q A V R P K	190
DIY-A	165	N L C S E A V K A A A A Y L R Q L E E E Q A V R P K	190

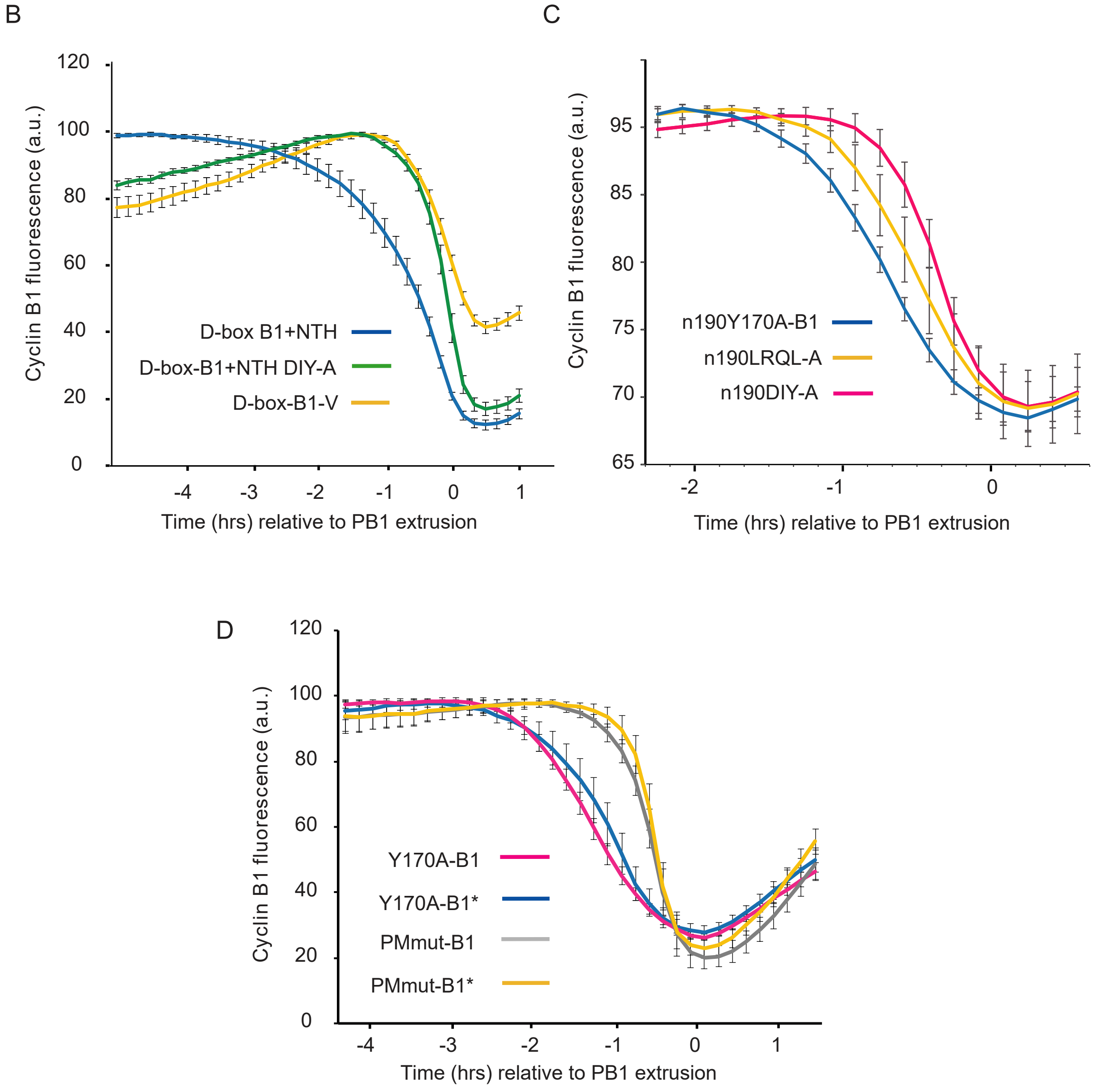

Figure S3

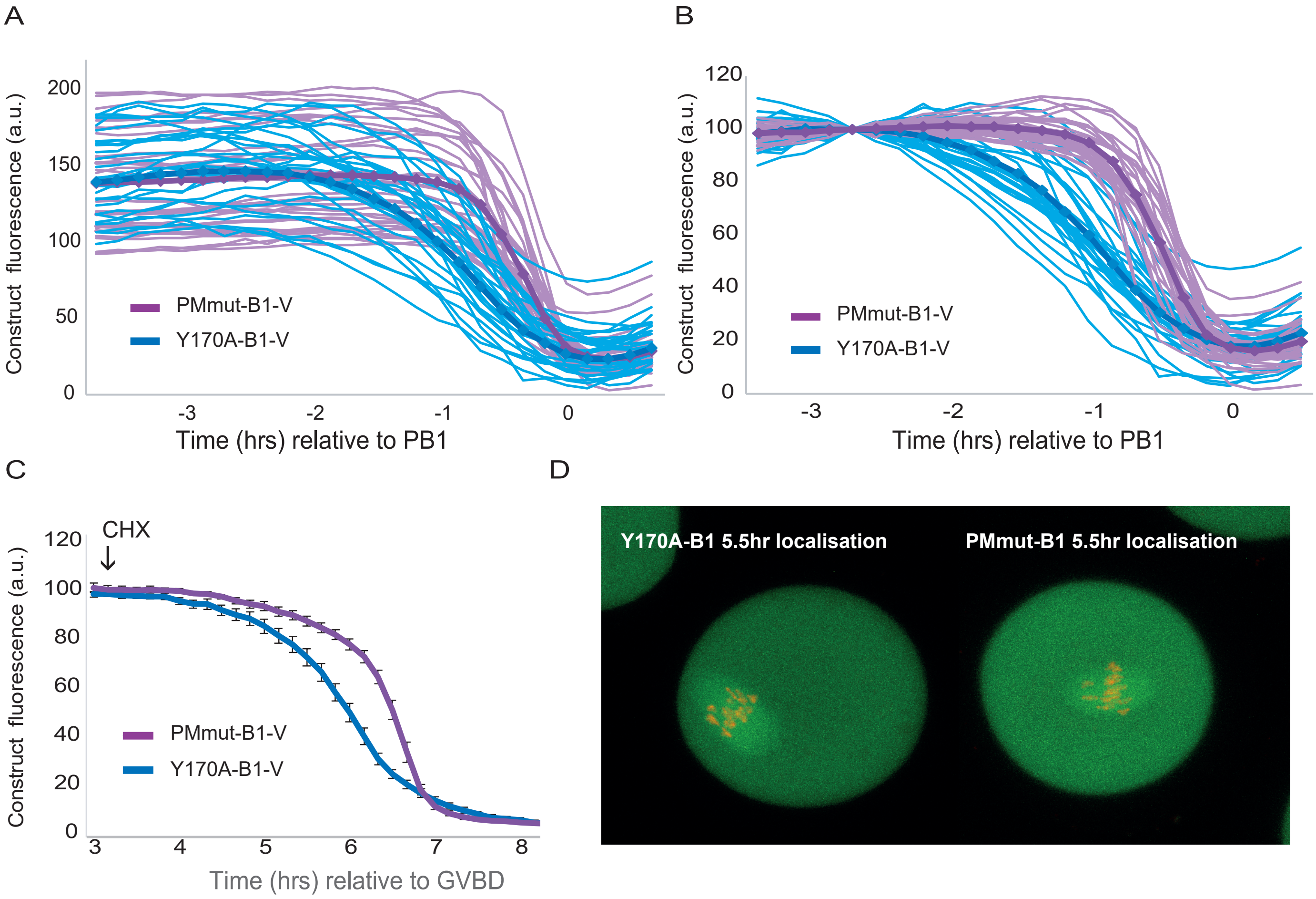
A
Construct fluorescence (a.u.)
200
150
100
50
0
PMmut-B1-V
Y170A-B1-V
-3
-2
-1
0
Time (hrs) relative to PB1
B
Construct fluorescence (a.u.)
120
100
80
60
40
20
0
PMmut-B1-V
Y170A-B1-V
-3
-2
-1
0
Time (hrs) relative to PB1
C
CHX
Construct fluorescence (a.u.)
120
100
80
60
40
20
0
PMmut-B1-V
Y170A-B1-V
3
4
5
6
7
8
Time (hrs) relative to GVBD
D
Y170A-B1 5.5hr localisation
PMmut-B1 5.5hr localisation

Figure S4

A

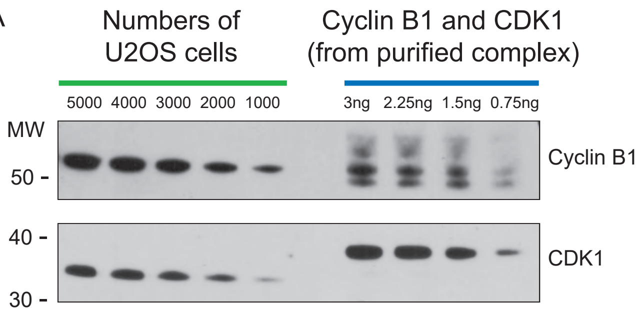

D

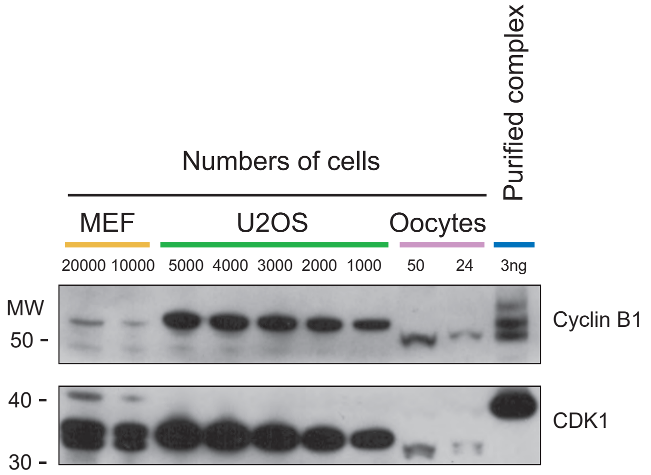

B

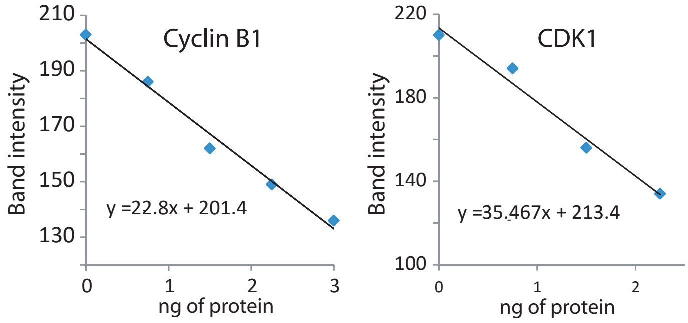

E

	Cyclin B1	CDK1
50 oocytes ~	815 U2OS cells	135 U2OS cells
1 oocyte ~	16.3 U2OS cells	2.7 U2OS cells
ng per single oocyte	11	1
nM	716	123
Ratio	6	1

C

U2OS cells	CDK1 band intensity	CDK1 in 1000 cells	Cyclin B1 band intensity	Cyclin B1 in 1000 cells
1000	203	*	185	0.72
2000	183	0.42	171	0.67
3000	171	0.40	156	0.66
4000	158	0.39	147	0.66
Average per 1000 cells		= 0.40 ng		= 0.68 ng

F

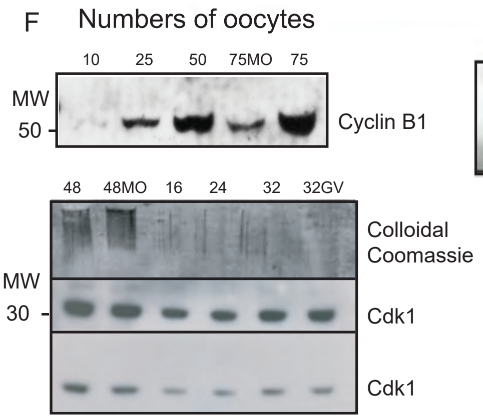

G

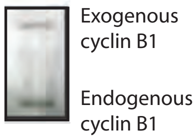

Figure S5

A

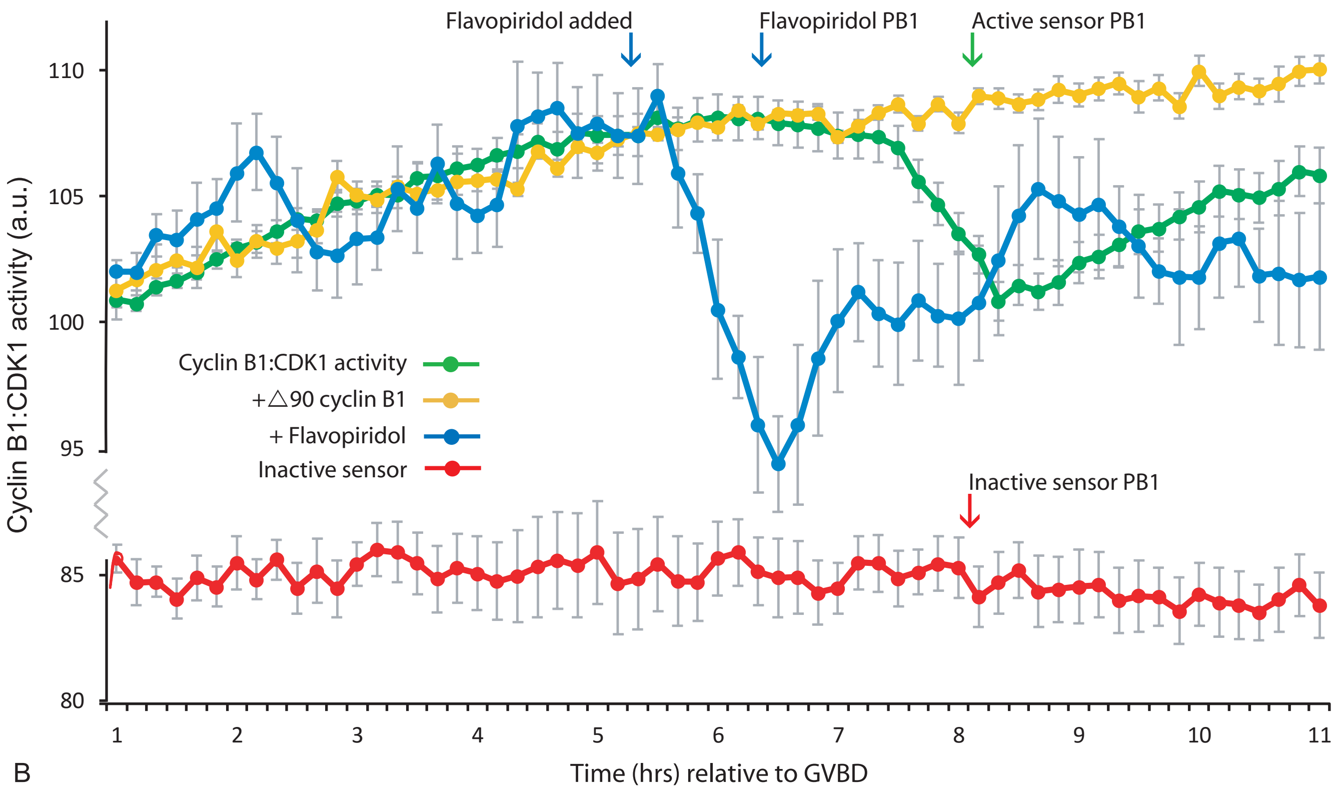
Flavopiridol added
Flavopiridol PB1
Active sensor PB1
Inactive sensor PB1
Cyclin B1:CDK1 activity (a.u.)
110
105
100
95
85
80
Cyclin B1:CDK1 activity
+△90 cyclin B1
+ Flavopiridol
Inactive sensor
1
2
3
4
5
6
7
8
9
10
11
Time (hrs) relative to GVBD

B

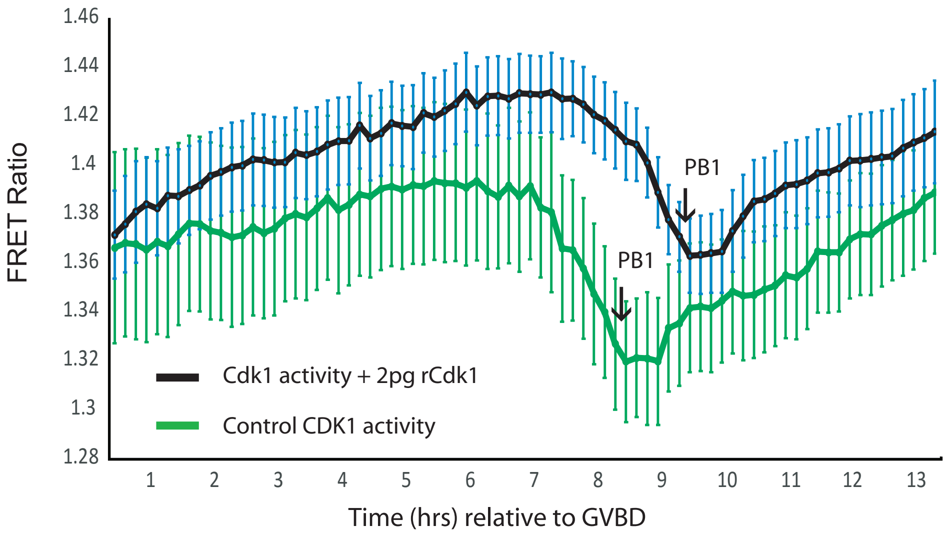
FRET Ratio
1.46
1.44
1.42
1.4
1.38
1.36
1.34
1.32
1.3
1.28
PB1
PB1
Cdk1 activity + 2pg rCdk1
Control CDK1 activity
1
2
3
4
5
6
7
8
9
10
11
12
13
Time (hrs) relative to GVBD

Figure S6

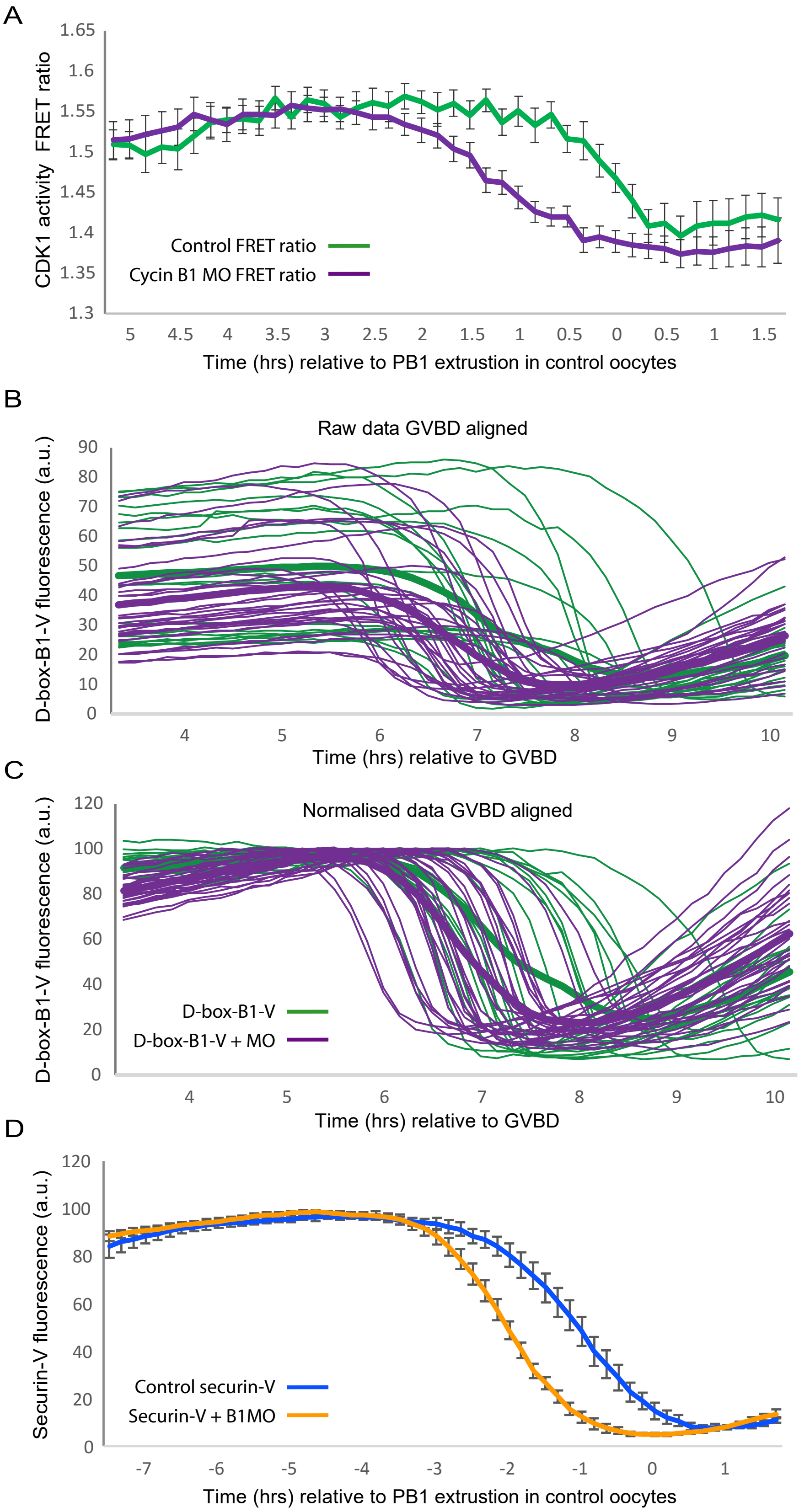
A
CDK1 activity FRET ratio
1.65
1.6
1.55
1.5
1.45
1.4
1.35
1.3
Control FRET ratio
Cycin B1 MO FRET ratio
5
4.5
4
3.5
3
2.5
2
1.5
1
0.5
0
0.5
1
1.5
Time (hrs) relative to PB1 extrusion in control oocytes
B
Raw data GVBD aligned
D-box-B1-V fluorescence (a.u.)
90
80
70
60
50
40
30
20
10
0
4
5
6
7
8
9
10
Time (hrs) relative to GVBD
C
Normalised data GVBD aligned
D-box-B1-V fluorescence (a.u.)
120
100
80
60
40
20
0
D-box-B1-V
D-box-B1-V + MO
4
5
6
7
8
9
10
Time (hrs) relative to GVBD
D
Securin-V fluorescence (a.u.)
120
100
80
60
40
20
0
Control securin-V
Securin-V + B1MO
-7
-6
-5
-4
-3
-2
-1
0
1
Time (hrs) relative to PB1 extrusion in control oocytes

Figure S7

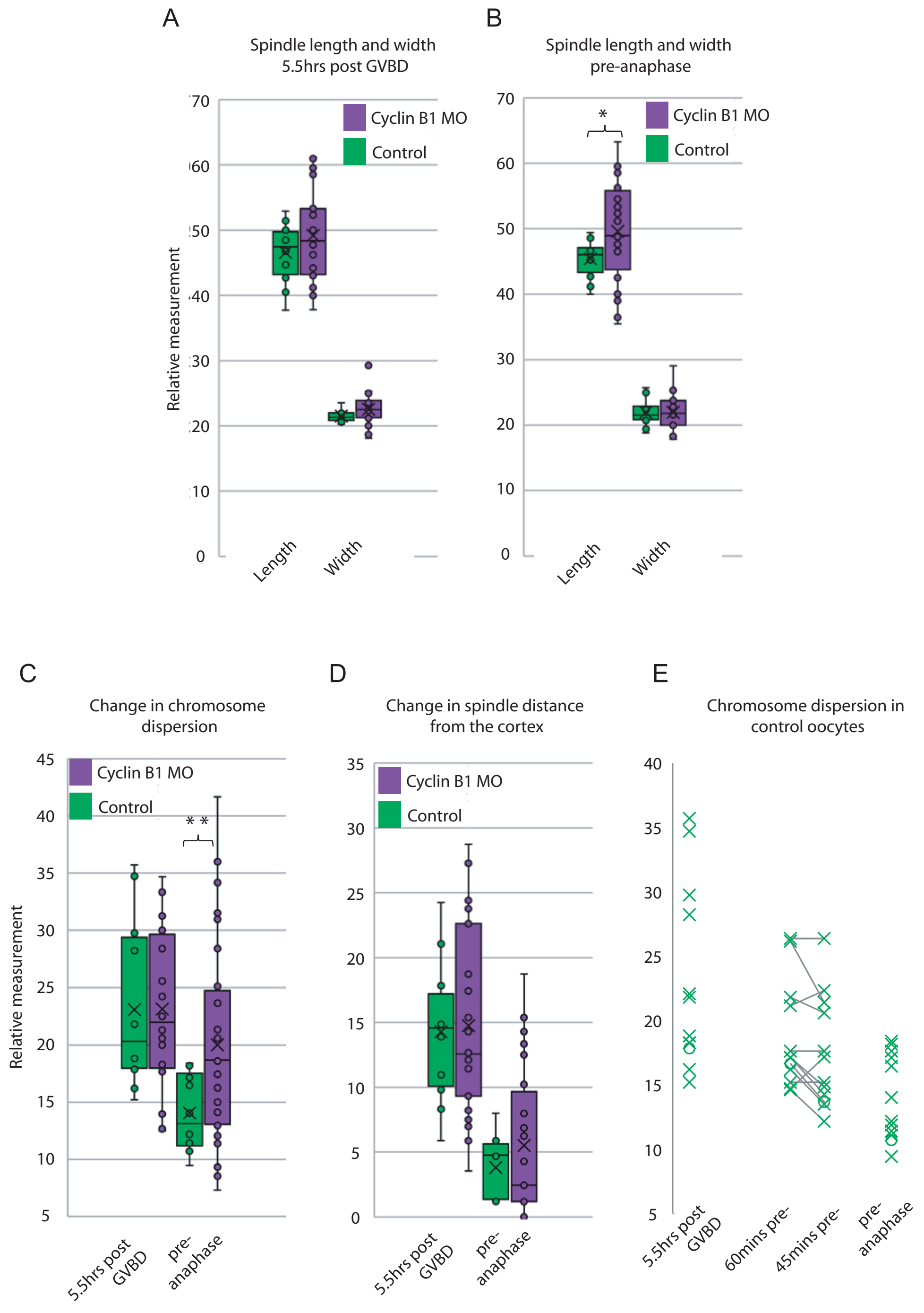
A
Spindle length and width
5.5hrs post GVBD
Relative measurement
70
60
50
40
30
20
10
0
Cyclin B1 MO
Control
Length
Width
B
Spindle length and width
pre-anaphase
*
C
Change in chromosome
dispersion
45
35
25
15
5
* *
5.5hrs post
GVBD
pre-
anaphase
D
Change in spindle distance
from the cortex
E
Chromosome dispersion in
control oocytes
60mins pre-
45mins pre-

Table S1

**Paired data comparisons between 5.5 hrs post GVBD and pre-anaphase; equality of means**			
Parameter	*Treatment*	*P value*	*Test*
Spindle length	Control	0.134	t-test (2-tailed)
Spindle length	Cyclin B1 MO	0.759	t-test (2-tailed)
Spindle width	Control	0.527	t-test (2-tailed)
Spindle width	Cyclin B1 MO	0.520	t-test (2-tailed)
Chromosome dispersion	Control	0.003	t-test (2-tailed)
Chromosome dispersion	Cyclin B1 MO	0.097	t-test (2-tailed)
Spindle distance from cortex	Control	0.002	Wilcoxon Signed Rank
Spindle distance from cortex	Cyclin B1 MO	0.000	Wilcoxon Signed Rank
