# Supplementary material for: Aneuploidy in Oocytes Is Prevented by Sustained CDK1 Activity through Degron Masking in Cyclin B1
Source: Dev Cell. 2019 Mar 11;48(5):672–684.e5. doi: 10.1016/j.devcel.2019.01.008 (PMC6416240; doi:10.1016/j.devcel.2019.01.008)
Supplement: Document S2. Article plus Supplemental Information [file mmc3.pdf]

Developmental Cell

Article

Aneuploidy in Oocytes Is Prevented by Sustained CDK1 Activity through Degron Masking in Cyclin B1

Graphical Abstract

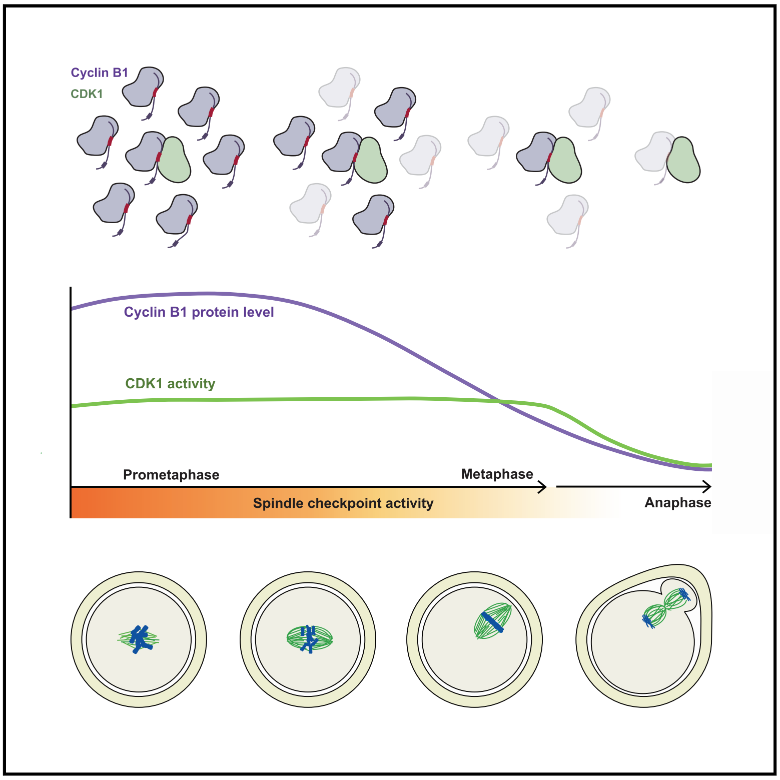

Highlights

- In mouse oocytes, an excess of cyclin B1 preserves CDK1 activity
- A motif in non-CDK1-bound cyclin B1 confers preferential APC/C targeting
- Non-CDK1-bound cyclin B1 is gradually destroyed before CDK1-bound cyclin B1
- Prolonged CDK1 activity assists the spindle checkpoint and prevents aneuploidy

Authors

Mark D. Levasseur, Christopher Thomas, Owen R. Davies, Jonathan M.G. Higgins, Suzanne Madgwick

Correspondence

suzanne.madgwick@newcastle.ac.uk

In Brief

The spindle checkpoint is often considered insufficient to prevent chromosome segregation errors in mammalian oocytes, yet mouse oocytes rarely divide with aneuploidies. Levasseur et al. elucidate how an excess of cyclin B1 preserves CDK1 activity to assist the spindle checkpoint to prevent segregation errors in mouse oocytes.

Levasseur et al., 2019, Developmental Cell *48*, 672–684
March 11, 2019 © 2019 The Authors. Published by Elsevier Inc.
https://doi.org/10.1016/j.devcel.2019.01.008

Aneuploidy in Oocytes Is Prevented by Sustained CDK1 Activity through Degron Masking in Cyclin B1

Mark D. Levasseur,[1,2] Christopher Thomas,[1,2,3] Owen R. Davies,[1] Jonathan M.G. Higgins,[1] and Suzanne Madgwick[1,4,*]
[1]Cell Division Biology Group, Institute for Cell and Molecular Biosciences, Faculty of Medical Sciences, Newcastle University, Newcastle upon Tyne, NE2 4HH, UK
[2]These authors contributed equally
[3]Present address: Max Planck Institute for Biophysical Chemistry, Am Fassberg 11, Gottingen 37077, Germany
[4]Lead Contact
*Correspondence: suzanne.madgwick@newcastle.ac.uk
https://doi.org/10.1016/j.devcel.2019.01.008

SUMMARY

Successful mitosis requires that cyclin B1:CDK1 kinase activity remains high until chromosomes are correctly aligned on the mitotic spindle. It has therefore been unclear why, in mammalian oocyte meiosis, cyclin B1 destruction begins before chromosome alignment is complete. Here, we resolve this paradox and show that mouse oocytes exploit an imbalance in the ratio of cyclin B1 to CDK1 to control CDK1 activity; early cyclin B1 destruction reflects the loss of an excess of non-CDK1-bound cyclin B1 in late prometaphase, while CDK1-bound cyclin B1 is destroyed only during metaphase. The ordered destruction of the two forms of cyclin B1 is brought about by a previously unidentified motif that is accessible in free cyclin B1 but masked when cyclin B1 is in complex with CDK1. This protects the CDK1-bound fraction from destruction in prometaphase, ensuring a period of prolonged CDK1 activity sufficient to achieve optimal chromosome alignment and prevent aneuploidy.

INTRODUCTION

CDK1 is the only cyclin-dependent kinase that is essential for the eukaryotic cell cycle (Santamaría et al., 2007). Throughout early mitosis, CDK1 is activated by its binding partner cyclin B1. Cyclin B1 must be maintained at a level sufficient to generate enough cyclin B1:CDK1 activity ("CDK1 activity") to drive the early stages of cell division (Gavet and Pines, 2010). By monitoring the status of kinetochore-microtubule attachments, spindle checkpoint proteins prevent the destruction of cyclin B1, thereby inhibiting anaphase until all chromosomes have congressed and their kinetochores have established stable attachments (Lara-Gonzalez et al., 2012). Thereafter, a sharp drop in CDK1 activity via cyclin B1 destruction is also important to drive the events of mitotic exit (Sullivan and Morgan, 2007).

The goal of the spindle checkpoint is to attenuate the activity of the anaphase-promoting complex or cyclosome (APC/C), an E3 ligase that directs the degradation of a number of cell cycle proteins. To ensure accurate passage through all stages of chromosome alignment and segregation, the APC/C must process its substrates in strict order, an order largely achieved via distinct substrate degradation motifs. The most well known of these motifs is the classic destruction motif, the D-box, that directs cyclin B1 destruction in metaphase (Glotzer et al., 1991).

In the absence of checkpoint activity, the APC/C and its co-activator Cdc20 form a bipartite co-receptor for D-box docking (He et al., 2013). This allows cyclin B1 to be ubiquitinated on multiple lysine residues and to be delivered to the 26S proteasome for destruction (Yamano et al., 1998). Prior to chromosome alignment, each unattached kinetochore generates a checkpoint signal, which is sufficient to prevent Cdc20 from binding to the D-box of metaphase substrates (Lara-Gonzalez et al., 2012). Chromosome misalignment in mitosis therefore strongly inhibits cyclin B1 destruction to prevent premature chromosome segregation and minimize the possibility of generating aneuploid daughter cells. Metaphase, the establishment of stable end-on kinetochore-microtubule attachments, coincides almost exactly with the destruction of cyclin B1, which is only initiated once the last chromosome aligns on the metaphase plate (Clute and Pines, 1999; Hagting et al., 2002).

Female mammalian meiosis is also driven by CDK1 activity (Ledan et al., 2001) and governed by the same checkpoint machinery as mitosis (Gorbsky, 2015). Yet, unlike mitosis, oocyte meiosis I (MI) is characterized by a lengthy period of cyclin B1 destruction, which initiates several hours ahead of metaphase. This destruction takes place before chromosome alignment is achieved, prior to the stabilization of kinetochore attachments, and while checkpoint proteins remain on kinetochores (Brunet et al., 1999; Davydenko et al., 2013; Gui and Homer, 2012; Kitajima et al., 2011; Lane and Jones, 2014; Lane et al., 2012; Nagaoka et al., 2011; Sebestova et al., 2012). Indeed, at the initiation of cyclin B1 destruction, nearly half of all mouse oocytes have chromosomes located away from the spindle equator (Lane et al., 2012).

Surprisingly, however, despite a prolonged period of cyclin B1 destruction ahead of metaphase I, the vast majority of mouse oocytes achieve chromosome alignment and undergo a division, which produces an egg with the correct complement of chromosomes. This situation raises two major questions: how does cyclin B1 evade the spindle checkpoint in oocytes and why does this early loss of cyclin B1 not result in eggs with higher frequencies of aneuploidy?

Here, we show that, in mouse oocytes, cyclin B1 is in excess of CDK1, the opposite of the situation in mitosis (Arooz et al., 2000).

 © 2019 The Authors. Published by Elsevier Inc.
This is an open access article under the CC BY license (http://creativecommons.org/licenses/by/4.0/).

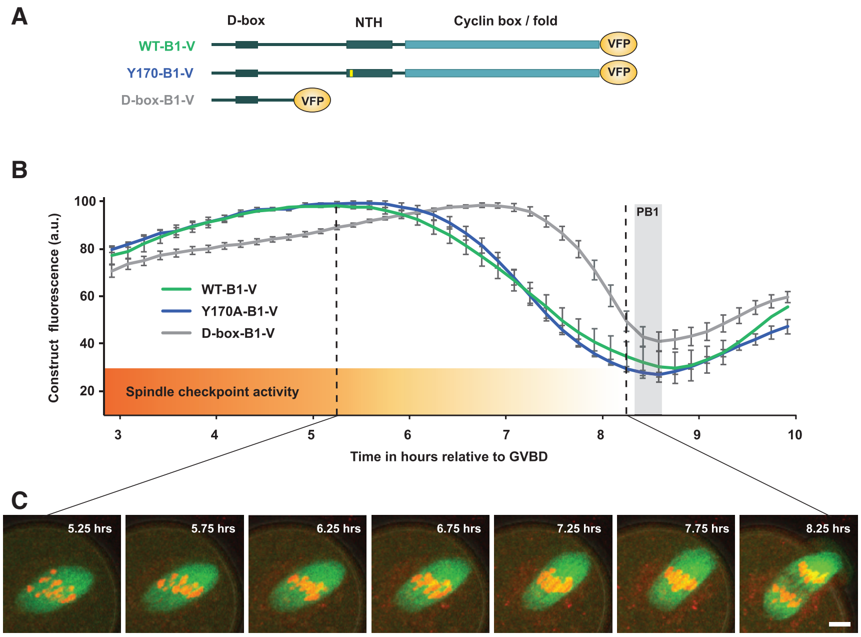

Figure 1. Cyclin B1 Destruction Begins ahead of Chromosome Alignment in MI Mouse Oocytes

(A) Schematic of cyclin B1 constructs: wild-type cyclin B1 (WT-B1-V), Y170A cyclin B1 (Y170A-B1-V), and the N-terminal 90 amino acids of cyclin B1 tagged with venus fluorescent protein (VFP) (D-box-B1-V).

(B) Mean destruction profiles of WT-B1-V (n = 16 oocytes), Y170A-B1-V (n = 62), and D-box-B1-V (n = 34) in maturing MI mouse oocytes. Error bars ± SEM. See also Figure S1.

(C) Representative images of maturing spindles at times indicated post GVBD in oocytes expressing Map7-GFP (microtubules, green) and SiR-DNA (DNA, red). Scale bar, 10 μm

This provides the oocyte with two pools of cyclin B1, which are then destroyed over different time periods: an excess of free cyclin B1, which is preferentially destroyed in late prometaphase, and a pool of CDK1-bound cyclin B1, which is preserved until metaphase. Known prometaphase APC/C substrates such as cyclin A2, which must be destroyed to allow passage into metaphase, contain motifs in addition to the D-box, which make their destruction less reliant on high levels of checkpoint-free Cdc20, thereby permitting them to escape full checkpoint inhibition (Di Fiore and Pines, 2010; Wolthuis et al., 2008; van Zon and Wolthuis, 2010; Boekhout and Wolthuis, 2015). Here, we uncover such a motif in cyclin B1. The location of this motif, masked within the cyclin B1:CDK1 interface, ensures that only free cyclin B1 is destroyed initially (when the checkpoint is active) while CDK1 activity is preserved. We show how this degron masking mechanism allows the activity of cyclin B1:CDK1 to be tightly regulated and to increase the fidelity of meiosis by allowing a prolonged period for chromosome alignment.

RESULTS

Cyclin B1 Destruction Initiates Ahead of Metaphase in MI Mouse Oocytes

To minimize the risk of aneuploidy, cyclin B1 destruction initiates at the onset of metaphase in mitosis. In contrast, cyclin B1 destruction initiates in prometaphase in MI mouse oocytes, approximately 3 h ahead of anaphase and before chromosomes have fully congressed. This seemingly precocious destruction of cyclin B1 has been considered to be erroneous. However, this is at odds with the low rates of aneuploidy observed in mouse oocytes.

To investigate how mouse oocytes continue to correct MI chromosome alignment despite falling levels of cyclin B1, we initially designed and tested two fluorescent non-CDK1-binding cyclin B1 constructs (Figure 1A). We expected that both would report the destruction timing of cyclin B1 without perturbing CDK1 activity. A non-CDK1-binding cyclin B1 reporter was important since, although fluorescent wild-type cyclin B1 expression (WT-B1-V) reports the initiation of endogenous cyclin B1 destruction (Reis et al., 2007), even moderate levels of over-expression can delay polar body (PB1) extrusion and increase the proportion of oocytes that arrest in MI. As expected, destruction of a full-length, non-CDK1-binding cyclin B1 reporter (Bentley et al., 2007; Goda et al., 2001) initiated in prometaphase, ahead of chromosome alignment, and generated a destruction profile almost identical to that of WT-B1-V (Figures 1B and 1C).

The D-box of Cyclin B1 Is Not Sufficient to Promote Prometaphase Cyclin B1 Destruction in MI

As in mitosis, the D-box is essential for all cyclin B1 degradation in mouse oocytes. We have previously demonstrated this in meiosis I and II for cyclin B1, which lacks the N-terminal 90 amino acids containing the D-box (Madgwick et al., 2004; Nixon et al., 2002; Herbert et al., 2003). Surprisingly, however, the D-box is not sufficient to generate a WT-B1 destruction profile. A truncated reporter that contained the canonical D-box sequence and lysine residues necessary for APC/C^{Cdc20} recognition and subsequent proteolysis (Yamano et al., 1998; Pines, 2011), was not targeted until 80–90 min after Y170A-B1-V (Figures 1B). This did not seem to be due to differences in protein synthesis rates or relative expression of the constructs (Figures S1A and S1B).

The initiation of D-box-B1-V destruction appeared to correlate with chromosome alignment (Figures 1A–1C), suggesting that D-box-B1-V targeting is inhibited by the spindle checkpoint to a greater extent than that of Y170A-B1-V. In support of this hypothesis, when oocytes were arrested in prometaphase with 100 nM nocodazole to stimulate a persistent checkpoint, gradual destruction of Y170A-B1-V was still permitted, yet D-box-B1-V destruction was prevented (Figure 2A). In contrast, where the spindle checkpoint was inhibited by the Mps1 inhibitor reversine, both constructs were degraded almost synchronously (Figure 2B), demonstrating that Y170A-B1-V is not simply a better target for ubiquitination by the APC/C but that the destruction of D-box-B1-V is restrained by the checkpoint to a far greater extent. Our observations in nocodazole imply that Y170A-B1-V destruction has a lower requirement for Cdc20

CellPress

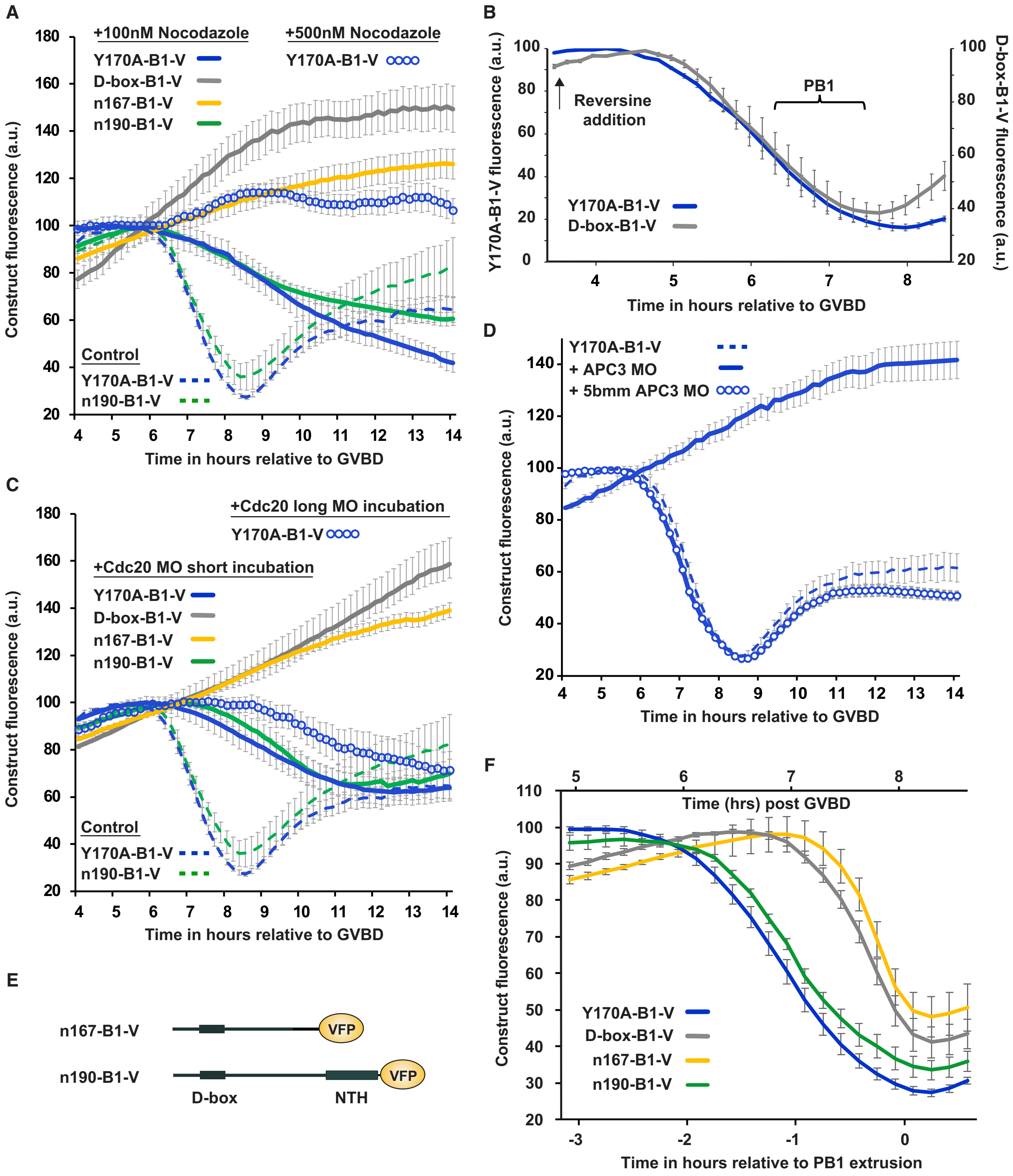

Figure 2. D-box-Only Recognition Is Not Sufficient for a Normal Cyclin B1 Destruction Profile

(A) Mean levels of Y170A-B1-V (n = 62 for 100 nM; n = 22 for 500 nM), D-box-B1-V (n = 31), n167-B1-V (n = 28), and n190-B1-V (n = 33) in MI oocytes following incubation in either 100-nM or 500-nM nocodazole as indicated. Control Y170A-B1-V and n190-B1-V are included as broken traces.

(legend continued on next page)

than D-box-B1-V. Indeed, when we prevented the prometaphase increase in Cdc20 levels with a morpholino oligo (Figure 2C; MO short incubation) the resultant expression profiles mimicked those in 100 nM nocodazole. As we have previously shown for WT-B1 after the same Cdc20 MO incubation, Y170A-B1-V destruction was still permitted (albeit at a reduced rate) despite lower Cdc20 levels (Reis et al., 2007). However, D-box-B1-V was stabilized. These data strongly suggest that the D-box-B1 reporter lacks regions that would otherwise permit a period of destruction in prometaphase.

A concentration of 100 nM nocodazole is the minimum required to inhibit anaphase and arrest oocytes in late prometaphase. Increasing the dose of nocodazole to 500 nM further stabilized Y170A-B1-V levels, demonstrating that Y170A-B1 destruction is responsive to the strength of the spindle checkpoint (Figure 2A). Similarly, increasing the length of the Cdc20 MO incubation (long MO incubation; Figure 2C) delayed Y170A-B1 destruction by an additional ~2 h. To further demonstrate the essential role of the APC/C^{cdc20} in both prometaphase and metaphase cyclin B1 destruction, we used an APC3 MO. The APC3 subunit is known to bind the IR motif in the tail of APC/C activators, and its depletion severely disrupts APC/C activity, resulting in the inhibition of cyclin A, cyclin B1, and securin degradation in mitosis (Izawa and Pines, 2011). In an analogous experiment, we incubated oocytes with an APC3 MO and found that all oocytes failed to degrade cyclin B1 following release from germinal vesicle (GV) arrest; this was not the case with a 5-base-mismatch MO (Figure 2D). This provides strong evidence that APC/C^{cdc20} activity is responsible for both early and late stages of cyclin B1 destruction.

A Second Destruction Motif Exists within the NTH of Cyclin B1 to Promote Destruction in Prometaphase

To determine the region of cyclin B1 necessary to recover prometaphase destruction, we made stepwise extensions to the C terminus of D-box-B1. The addition of residues between 167 and 190 restored prometaphase destruction (Figures 2E and 2F; compare n167-B1-V and n190-B1-V). Furthermore, when oocytes were arrested in prometaphase with the use of 100-nM nocodazole, or when Cdc20 levels were knocked down, n190-B1-V remained a destruction target like Y170A-B1-V, while n167-B1-V was stabilized like D-box-B1-V (Figures 2A and 2C).

Between residues 167 and 190 lies the N-terminal helix (NTH) of cyclin B1 (residues 170–196; Figure 3A), an integral part of the CDK1 binding interface (Brown et al., 2015). Fusing the NTH to the D-box-B1 (D-box-B1+NTH) fully rescued a Y170A-B1-like destruction profile, effectively converting D-box-B1 to a prometaphase destruction target (Figures 3B, 3C, and S2B), demonstrating that the critical region lies within the NTH. To identify residues necessary for prometaphase targeting, we made a series of point mutations in the D-box-B1+NTH and n190-B1 reporters and found specific mutations that delayed the destruction of the expressed protein by ~60–80 min (Figure S2). This mutagenesis revealed 7 residues, DIY (173–175) and LRQL (178–181), which we suggest constitute a novel motif (hereafter, named the ProMetaphase [PM] motif) able to direct APC/C-mediated proteolysis of free cyclin B1 in late prometaphase. Indeed, mutation of these 7 residues in Y170A-B1-V generated a full-length cyclin B1 mutant (PMmutB1-V; Figure 3A), which was now destroyed at the same time as D-box-B1-V (Figures 3B, 3C, and S2D). Further evidence that the PM motif confers prometaphase destruction was provided by the stability of PMmut-B1 protein in the presence of nocodazole (Figure 3D).

The distinct order of Y170A-B1-V and PMmut-B1-V destruction targeting was not an artefact of dissimilar levels of exogenous expression nor a result of differences in translation efficiency (Figures S3A–S3C and S4G). Furthermore, confocal microscope scanning through the MI spindle of oocytes expressing PMmutB1-V and Y170A-B1-V demonstrated no discernible difference in localization, despite a clear shift in the timing of destruction targeting (Figure S3D). Together, these studies identify a PM motif within the NTH of cyclin B1 that leads to initiation of its destruction in prometaphase.

We have referred to the sequence we have identified as an important signaling "motif" rather than a degron since the term degron is used inconsistently within the literature (Guharoy et al., 2016). However, it is worth noting that the PM motif also fits an established definition of a degron: a protein element that confers instability, important in the regulation of protein degradation rates (Varshavsky, 1991).

Cyclin B1 Levels Are in Excess and Do Not Report CDK1 Activity in Mouse Oocytes

The location of the PM motif in cyclin B1 is potentially significant given that the NTH is an integral part of the CDK1 binding interface (Figure 4A; Brown et al., 2015) and in context of the ratio of cyclin B1 to CDK1 in mouse oocytes. Previously, cyclin B1 has been reported to exist in excess of CDK1 in prophase mouse oocytes, the opposite of the situation in mitosis, where CDK1 is in excess of cyclin B1 (Arooz et al., 2000). We confirmed this finding in late prometaphase oocytes by immunoblotting and found cyclin B1 to be in 6-fold excess over CDK1 (Figures S4A–S4E). Consequently, it is expected that there will be a large pool of non-CDK1-bound cyclin B1 in prometaphase mouse oocytes.

The position of the PM motif within the NTH suggests how the cellular destruction machinery is able to discriminate between CDK1-bound and free cyclin B1. We propose that early cyclin

(B) Mean destruction profiles of Y170A-B1-V (n = 20) and D-box-B1-V (n = 17) on addition of reversine to inhibit checkpoint activity. Traces are aligned to the addition of reversine at 3.5 h post GVBD. PB1s emerged over the period shown.

(C) Mean levels of Y170A-B1-V (n = 38 for short incubation; n = 20 for long incubation), D-box-B1-V (n = 20), n167-B1-V (n = 20), and n190-B1-V (n = 20) in oocytes following either a short (1.5–2 h) or a long (6–7 h) Cdc20 MO incubation period. Note that oocytes do not extrude a PB1 in either dose of nocodazole or after either Cdc20 MO incubation period.

(D) Mean levels of Y170A-B1-V in untreated oocytes (n = 62) or in oocytes released from prophase arrest 6 h after an injection of either an APC3 MO (n = 18) or an APC3 5-base pair-mismatch MO (5-bp mm; n = 25).

(E) Schematic of cyclin B1 truncations: n167-B1-V and n190-B1-V, the N-terminal 167 and 190 amino acids of cyclin B1 tagged with VFP.

(F) Mean destruction profiles Y170A-B1-V (n = 62), D-box-B1-V (n = 34), n167-B1-V (n = 38), and n190-B1-V (n = 38) in maturing MI oocytes.

Error bars ± SEM throughout.

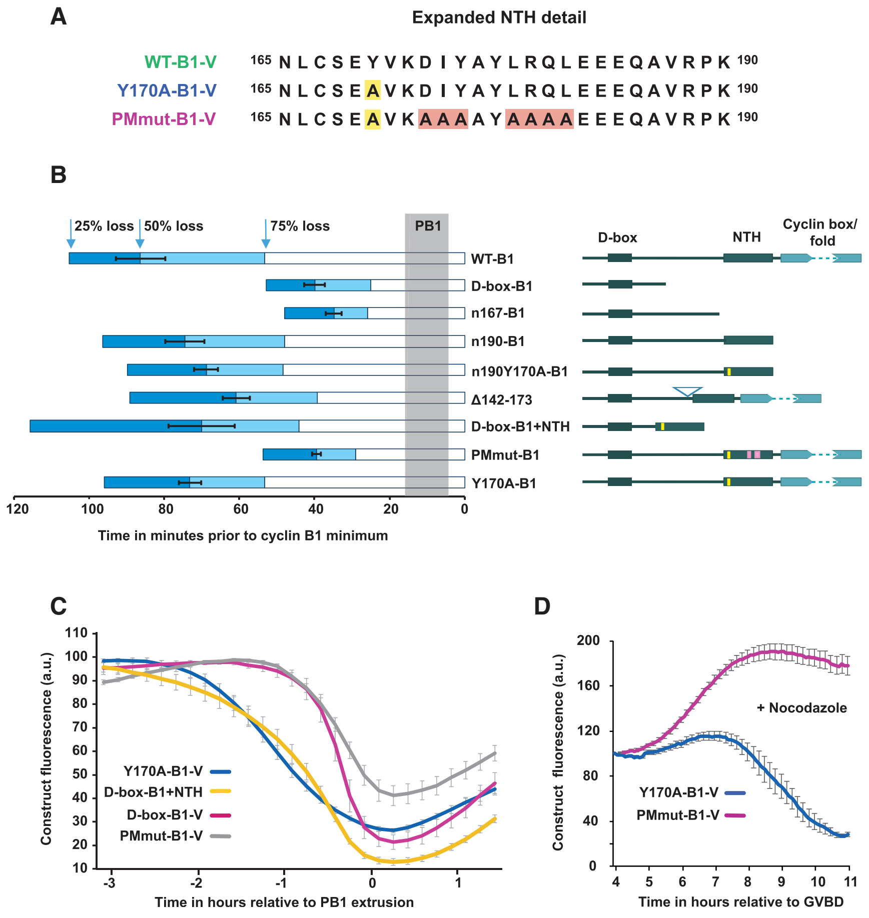

Figure 3. A Second Destruction Motif within the NTH of Cyclin B1 Promotes Prometaphase Cyclin B1 Destruction

(A) NTH sequence detail in WT-B1-V, Y170A-B1-V, and a PM-motif mutation (PMmut-B1-V). Yellow and pink boxes relate to sequence changes highlighted in (B).

(B) Destruction timing of cyclin B1 truncations and mutants. Other than WT-B1-V, all lack the ability to bind CDK1 and act as reporters of destruction timing without perturbing endogenous CDK1 activity. Schematic representations of constructs are shown to the right (yellow and pink marks denote sequence changes highlighted in A). The length of each bar to the left indicates destruction timings in minutes, where time 0 = maximal destruction. PB1s were extruded over the period shaded in gray. Open white bars indicate the timing of 75% protein destruction. The light blue extensions indicate 50% protein destruction, and dark blue indicates 25% protein destruction.

(C) Mean Y170A-B1-V, D-box-B1-V, D-box-B1+NTH (n = 22), and PMmut-B1-V (n = 32) destruction profiles in maturing MI oocytes (See Figure S2 for destruction profiles of additional truncations and mutants).

(D) Mean levels of Y170A-B1-V (n = 12) and PMmut-B1-V (n = 28) in MI oocytes following incubation in 100 nM nocodazole.

Error bars ± SEM throughout.

B1 destruction represents primarily the loss of free, non-CDK1-bound cyclin B1, targeted in prometaphase via its D-box and PM motif, a situation that is mimicked by the non-CDK1-binding Y170A-B1 mutant. However, since only the D-box is accessible in CDK1-bound cyclin B1, bound cyclin B1 destruction is initially prevented, a situation that is mimicked by the D-box-B1 or PMmut-B1 reporter. This led us to predict that CDK1 activity remains elevated during prometaphase destruction of free cyclin B1 and falls only later when CDK1-bound cyclin B1 is destroyed.

To test this hypothesis, we used a real-time live assay based on the CDK1 fluorescence resonance energy transfer (FRET) biosensor first developed for use in mitosis (Gavet and Pines, 2010). After validating the sensor for use in oocytes (Figure S5), we found that CDK1 activity was remarkably stable through prometaphase. During the first ~1.5 h of cyclin B1 destruction, CDK1 activity was preserved, declining only in the final hour before PB1 extrusion as a result of D-box directed cyclin B1 destruction (Figure 4B). Indeed, though metaphase is not coupled to the initiation of cyclin B1 destruction in oocytes, the decline in CDK1 activity closely coincides with the time at which stable end-on kinetochore-microtubule attachments are formed and checkpoint proteins are maximally depleted from kinetochores (Kitajima et al., 2011; Lane et al., 2012). We suggest that this prolonged plateau of CDK1 activity explains how oocytes are able to continue to align their chromosomes despite the early loss of cyclin B1.

CDK1 Binding Protects Cyclin B1 from Destruction in Prometaphase

To demonstrate that CDK1 binding protects cyclin B1 from destruction, we altered the ratio of CDK1 to cyclin B1 by overexpressing CDK1. We made use of kinase-dead CDK1 (Leroy et al., 1996) to allow us to assess PM motif masking independent of the prolonged increase in CDK1 activity, which would be generated by adding wild-type CDK1.

Prophase-arrested oocytes were injected with K33ACDK1-C (cerulean labeled K33ACDK1) cRNA to generate CDK1 protein levels in excess of cyclin B1 by 5.5 h post germinal vesicle break down (GVBD). Oocytes were then allowed to mature after receiving a second injection of either WT-B1-V or Y170A-B1-V cRNA. Importantly, WT-B1-V and Y170A-B1-V fluorescence increased at the same rate in K33ACDK1-C overexpressing oocytes.

As expected, K33ACDK1 had a dominant-negative effect, which resulted in delayed targeting of both fluorescent cyclin B1 proteins and failure to extrude polar bodies. Critically, however, once targeted, the rate of Y170A-B1 protein destruction was unperturbed in the presence of K33ACDK1-C (Figures 4C and 4D). In contrast, WT-B1 destruction was significantly slower in oocytes expressing K33ACDK1-C (Figures 4C and 4D). Indeed, WT-B1-V levels failed to reach a minimum > 4 h after Y170A-B1-V had undergone complete destruction. We would not expect K33ACDK1-C to fully protect wild-type cyclin B1 since dynamic exchange ensures repetitive dissociation of cyclin B1 from CDK1, periodically revealing the PM motif. However, in Y170A-B1, which cannot bind CDK1, the PM motif is

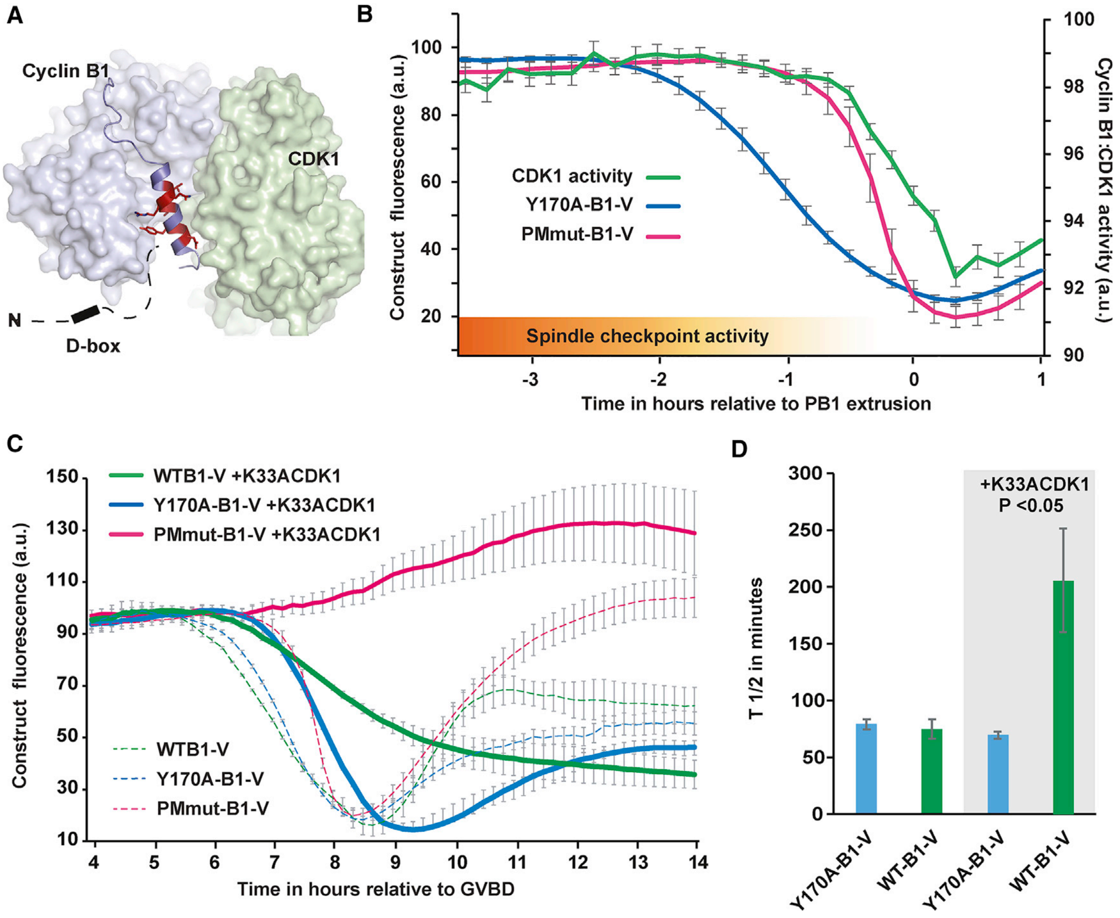

Figure 4. An Excess of Free Cyclin B1 Is Destroyed ahead of CDK1-Bound Cyclin B1; Therefore, Total Cyclin B1 Levels Do Not Reflect CDK1 Activity in MI Mouse

(A) Surface representation of cyclin B1:CDK1 from the crystal structure of its complex with CKS2 (pdb accession 4Y72); (Brown et al., 2015). The NTH and preceding loop are excluded from the surface, and their backbones are instead shown (in blue), with residues 173-DIY-175 and 178-LRQL-181 highlighted in red. The flexible N-terminal extension harboring the D-box is illustrated in a black dotted line.

(B) Mean destruction profiles of Y170A-B1-V and PMmut-B1-V alongside CDK1 activity determined using a FRET biosensor (n = 72). The decrease in spindle checkpoint activity is illustrated in an orange gradient.

(C) Mean WT-B1-V (n = 18), Y170A-B1-V (n = 15), and PMmut-B1-V (n = 31) destruction traces following overexpression of K33ACDK1-C (kinase dead CDK1). Control, non-K33ACDK1-C expressing destruction profiles are included as broken traces. Note that oocytes do not extrude polar bodies, presumably because of a dominant-negative effect of K33ACDK1-C.

(D) Rates of WT-B1-V and Y170A-B1-V destruction measured as $T_{1/2}$ in min in oocytes with and without K33ACDK1-C expression.

Error bars ± SEM throughout.

always accessible, and accordingly, its destruction profile was unperturbed. We reasoned that the profile of endogenous cyclin B1 destruction would be like that of WT-B1-V, explaining why K33ACDK1-C expressing oocytes were unable to extrude a PB.

In a further experiment, K33ACDK1-C expressing oocytes received a second injection of PMmut-B1-V. Though PMmut-B1 protein was unable to bind CDK1 (due to the absence of residues critical for CDK1 binding), this protein was not a destruction target and instead remained stable for a number of hours post GVBD, despite possessing a D-box. This result was explained using confocal microscopy, where we found that > 90% of all K33ACDK1-C-expressing oocytes arrested with misaligned chromosomes and short immature spindles (data not shown). We reasoned that oocytes overexpressing K33ACDK1-C do not reach the stage of MI development at which D-box-only cyclin B1 destruction is initiated. We conclude that, prior to the establishment of a metaphase spindle, the APC/C has a clear preference for non-CDK1-bound cyclin B1 owing to the exposure of the PM motif.

Limiting the Excess of Cyclin B1 Results in Premature MI Exit

Our data suggest the existence of a novel cyclin B1 motif that is able to promote the destruction of an excess of non-CDK1-bound cyclin B1 ahead of CDK1-bound cyclin B1. While this mechanism has clear implications for our understanding of cell cycle regulation in mouse oocytes, we wished to know whether the excess of cyclin B1 plays an important role in oocyte biology.

To test this, we knocked down cyclin B1 levels (cyclin B1 MO; Figure S4F) such that oocytes contained a ~2-fold rather than

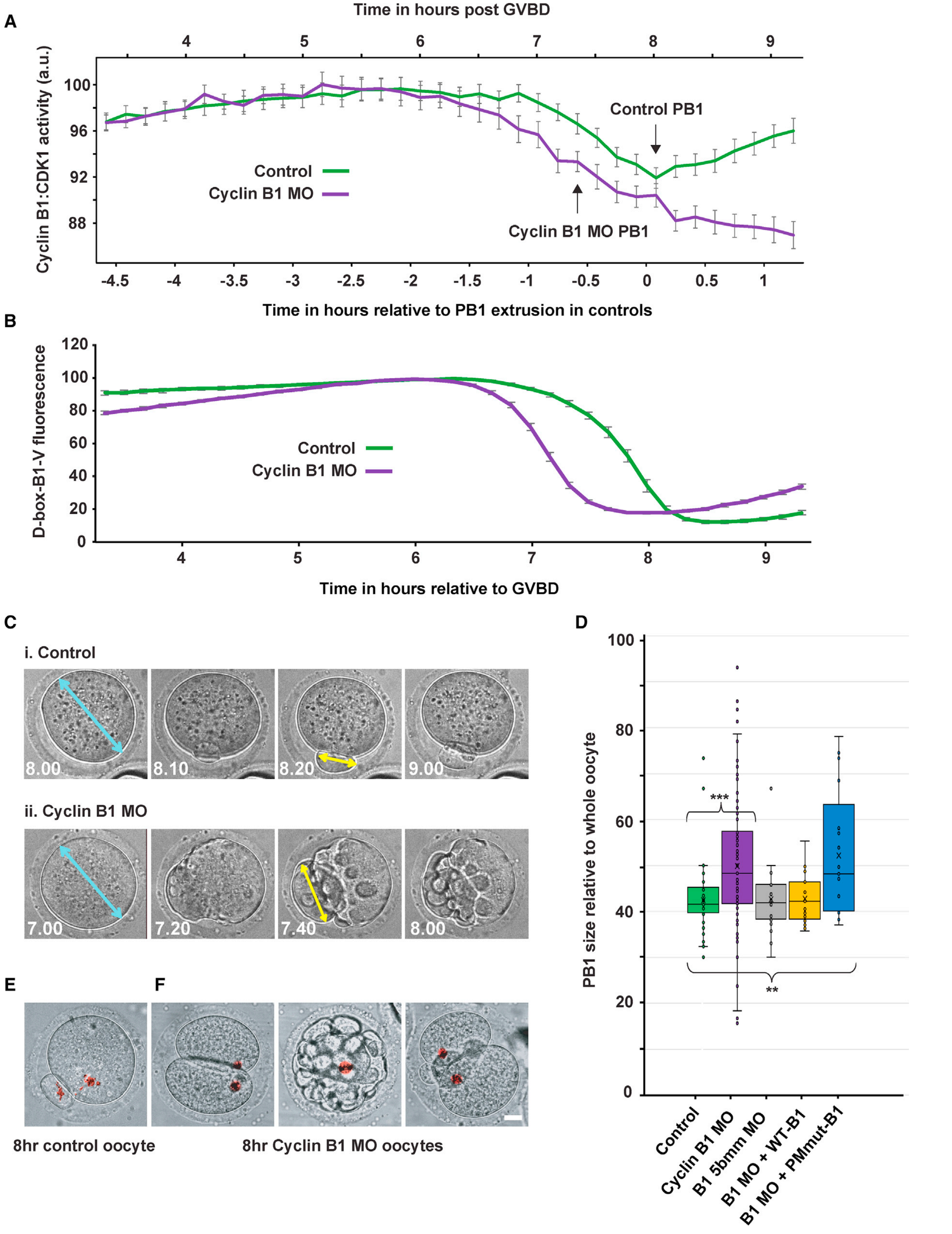

(legend on next page)

~6-fold excess of cyclin B1 at 5.5 h post GVBD (cyclin B1 MO oocytes). Importantly, given that cyclin B1 still remained in excess of CDK1, we observed no difference in the gradual increase in CDK1 activity between control and cyclin B1 MO oocytes. However, cyclin B1 MO oocytes then failed to maintain a plateau of CDK1 activity and exited MI earlier (Figures 5A and S6A). In addition, the D-box-B1 reporter was also destroyed earlier in cyclin B1 MO oocytes (Figures 5B, S6B, and S6C), strongly suggesting that early MI exit is due to premature destruction of CDK1-bound cyclin B1 via the D-box. Given this, it seemed plausible that depletion of the excess of cyclin B1 in cyclin B1 MO oocytes reduced overall APC^{cdc20} substrate competition, likely perturbing the destruction dynamics of other cell cycle proteins. Indeed, we found that the APC^{cdc20} D-box substrate securin was also targeted earlier in cyclin B1 MO oocytes (Figure S6D).

Cyclin B1 MO oocytes attempted to extrude a PB1 ~40 min earlier than control oocytes; however, the extruded body was often enlarged and accompanied by excessive membrane blebbing not normally seen in oocytes (Figures 5C–5F). This phenotype was not observed with a control 5-base-mismatch MO, or if MO treated oocytes were subsequently rescued by microinjection of WT-B1-V (B1 MO + WT-B1), where chromosomes condensed normally and segregated between the oocyte and PB1 with normal morphology. In contrast, where PMmut-B1-V was added back to B1 MO treated oocytes in place of WT-B1-V (B1 MO + PMmut-B1), the MO phenotype was not rescued; instead, 65% of oocytes arrested ahead of PB1 extrusion and the remainder often still extruded an enlarged PB1 (Figure 5D).

To explore the cyclin B1 MO phenotype, control and cyclin B1 MO oocytes were injected with Map7-GFP (Bulinski et al., 1999) cRNA and incubated in the DNA dye SiR-DNA to monitor microtubules and chromosomes, respectively. Live fluorescence time-lapse imaging was performed through multiple z-sections from ~4 to 12 h post GVBD. Using this method, the majority of control oocytes passed through MI with normal timing (83%). However, in stark contrast, a number of grossly abnormal phenotypes were observed in dividing cyclin B1 MO oocytes, including enlarged or multiple polar bodies and failure of anaphase and/or cytokinesis (Figures 6A and 6B; Video S1).

We reasoned that, because CDK1 activity was similar over the first 5–6 h (Figure 5A), the spindles of cyclin B1 MO oocytes would likely not differ from control oocytes at 5.5 h post GVBD. Indeed, for all parameters measured (Figure 6C), no significant differences were detected between control and cyclin B1 MO groups at 5.5 h post GVBD (Figures 6D, S7A, S7C, and S7D; Table 1). Furthermore, in agreement with prior work (Lane et al., 2012; Kitajima et al., 2011; Sebestova et al., 2012), at this time point, misaligned chromosomes were readily observed, and the spindle had not yet fully migrated to the cortex, even though cyclin B1 destruction was already underway. Subsequently, however, while control oocytes continued to align their chromosomes and relocate their spindles, cyclin B1 MO oocytes failed to significantly reduce the area over which chromosomes were dispersed, suggesting they were unable to promote further chromosome congression (second plot, Figures 6D and S7C).

In addition, pre-anaphase measurements taken of spindle length and spindle distance from the cortex were significantly more varied in cyclin B1 MO oocytes than in control oocytes at the same stage (Figures 6D and S7B–S7D; Table S1). Therefore, inefficient spindle maintenance and misregulation of chromosome alignment due to the precocious loss of CDK1 activity in late prometaphase appeared to be a direct result of a depleted "buffer" of cyclin B1.

DISCUSSION

Compared to prometaphase in mitosis, prometaphase I in mammalian oocytes is an exceptionally long and complicated process; pairs of homologous chromosomes must align on a bipolar spindle formed in the absence of functional centrosomes, a process that involves multiple rounds of error correction, and there are significant delays between each correction attempt and a lengthy period of spindle migration (Clift and Schuh, 2015; Holubcová et al., 2015; Kitajima et al., 2011; Verlhac et al., 2000). Even when a robust checkpoint is maximally activated in mitosis, its ability to delay exit is not indefinite, and mitotic cells are still susceptible to "slippage" caused by slow degradation of cyclin B1 (Brito and Rieder, 2006). Furthermore, checkpoint signaling may be problematic in larger cells where the ratio of kinetochores to cell volume is low (Galli and Morgan, 2016), a problem likely to be significant in an oocyte that is typically ~300 times the volume of a mitotic cell. It is perhaps not surprising that a few erroneous kinetochore-microtubule attachments are not sufficient to inhibit anaphase in MI oocytes (Lane et al., 2012; Sebestova et al., 2012; Gui and Homer, 2012; Nagaoka et al., 2011).

As a result, cyclin B1:CDK1 activity is potentially vulnerable to premature proteolysis for a number of hours in MI oocytes. Indeed, in mouse oocytes, misaligned bivalents are a common

Figure 5. Restricting the Excess of Cyclin B1 Causes Premature D-box Only Mediated Destruction, Severely Compromising MI Division

(A) CDK1 activity in untreated (n = 72) and cyclin B1 MO injected oocytes (n = 64). Time 0 = PB1 extrusion in control oocytes while cyclin B1 MO oocytes extrude aberrant PB1's ~40 min earlier as indicated. Upper x axis indicates timing post GVBD for the mean FRET trace within each treatment group.

(B) Mean destruction profile of D-box-B1-V in untreated (n = 20) and cyclin B1 MO oocytes (n = 31). GVBD to PB1 extrusion = 8 h and 5 min (± 5 min) in control oocytes and 7 h and 24 min (± 11 min) in cyclin B1 MO oocytes.

(C) Representative images of (i) PB1 extrusion in a control oocyte and (ii) attempted PB1 extrusion in a cyclin B1 MO oocyte at times indicated post GVBD. For comparison, the size of each PB1 was recorded as a proportion of the diameter of the oocyte prior to its extrusion (length of the yellow arrow as a percentage of the blue arrow).

(D) PB1 size in control oocytes (green, n = 39), cyclin B1 MO oocytes (purple, n = 64), oocytes microinjected with a control cyclin B1 5-base-mismatch MO (B1 5-bpmm MO; gray, n = 20), and cyclin B1 MO oocytes subsequently rescued with WT-B1-V (yellow, n = 21). 5-base-mismatch MO oocytes and WT-B1-V rescue oocytes divided normally and extruded PBs without the excessive blebbing noted in MO oocytes. The 5th box represents the size of the PB1 extruded in cyclin B1 MO oocytes subsequently injected with PMmut-B1-V (blue, n = 17). However, the majority of these oocytes (31/48) did not extrude a PB1 and instead arrested in either metaphase or early anaphase. Error bars ± SEM throughout. *** denotes p value of < 0.001; ** denotes p value of < 0.005.

(E and F) Further examples of (E) a control oocyte and (F) cyclin B1 MO oocytes 8 h post GVBD (Hoechst DNA staining in red). Scale bar, 10 μm.

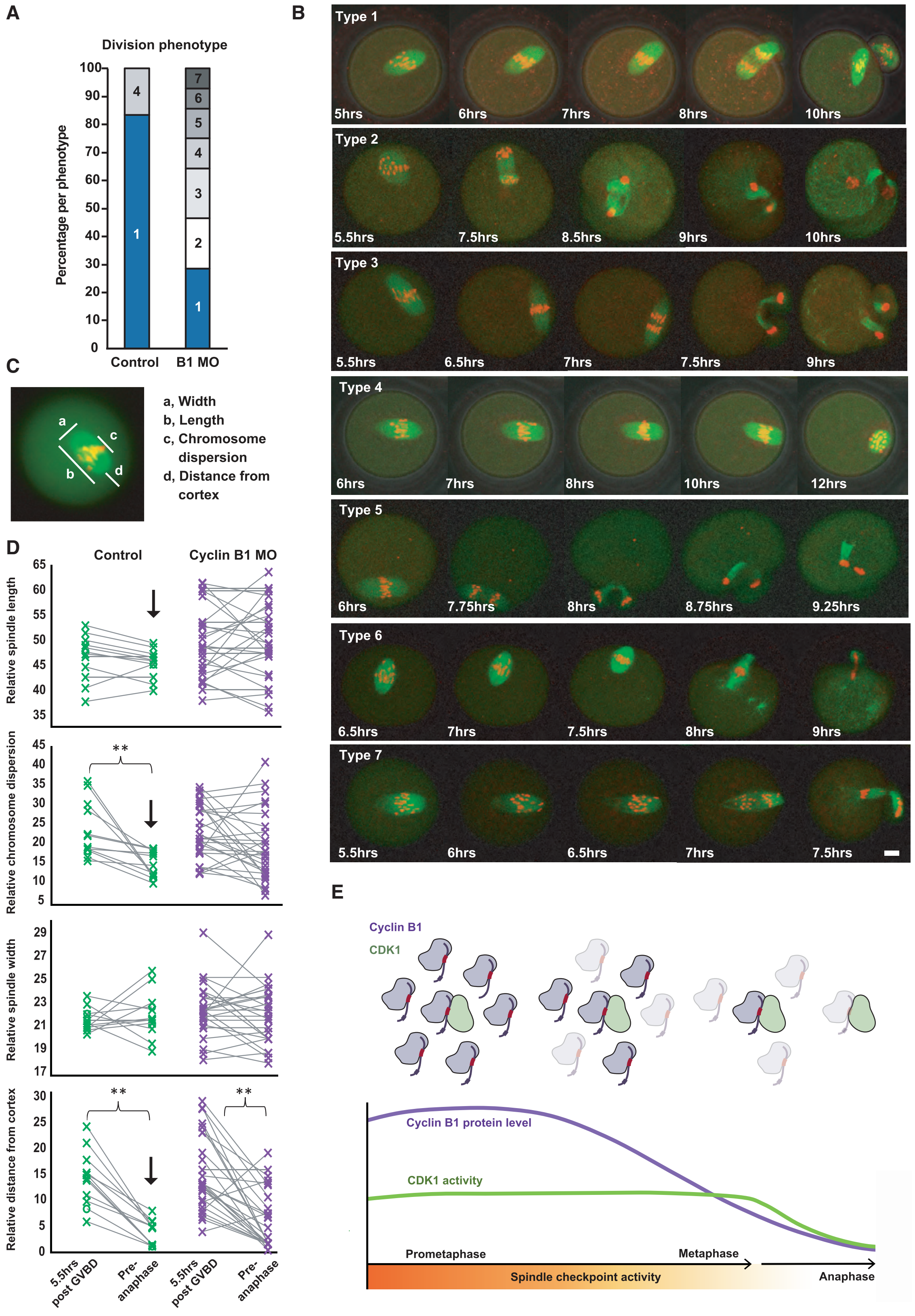

(legend on next page)

Table 1. Significance of Differences in Variance and Means between Control and Cyclin B1 MO Datasets

Independent Sample Comparisons between Control and Cyclin B1 MO Oocytes; Variance and Mean

Parameter	Time Point	Variance p Value	Mean p Value
Spindle length	5.5 h post GVBD	0.059	0.248
Spindle width	5.5 h post GVBD	0.066	0.135
Chromosome dispersion	5.5 h post GVBD	0.932	0.981
Spindle distance from cortex	5.5 h post GVBD	0.110	0.893
Spindle length	15 min pre-anaphase	0.003	0.015
Spindle width	15 min pre-anaphase	0.285	0.984
Chromosome dispersion	15 min pre-anaphase	0.016	0.004
Spindle distance from cortex	15 min pre-anaphase	0.004	0.309

Control and cyclin B1 MO oocyte spindle lengths, widths, chromosome dispersion distances, and spindle cortex distances were judged to be of equivalent variance at 5.5 h post GVBD. At this same time point, mean values were not significantly different from one another. At the later time point of 15 min prior to anaphase, no differences were detected in spindle widths between control and cyclin B1 MO oocytes. However, spindle lengths, chromosome dispersion distances, and spindle cortex distances were significantly more varied in cyclin B1 MO oocytes than in control oocytes. Furthermore, spindles were significantly longer, and chromosomes were spread over a significantly greater distance in cyclin B1 MO oocytes when compared to control oocytes.

feature up to 2 h after the initiation of cyclin B1 degradation (Lane et al., 2012). High-resolution imaging determines an average of two failed chromosome biorientation attempts per oocyte between 5 and 6 h post GVDB (Kitajima et al., 2011). We also often find that chromosomes do not fully congress until 1 h before anaphase when cyclin B1 is already depleted by >50% (Figures 1B, 1C, and S7E).

However, while this seemingly precocious destruction of cyclin B1 might appear detrimental, almost all chromosomes achieve biorientation before anaphase, and the incidence of aneuploidy in mouse oocytes is a mere 2%–4% (Lane et al., 2012; Pan et al., 2008; Homer et al., 2005). Here, we provide a model that resolves this puzzle by demonstrating that CDK1 activity is preserved in mouse oocytes despite the loss of cyclin B1. Our results reveal that a surplus of free cyclin B1 exists, and we propose that this acts as a protective buffer throughout the prolonged duration of MI spindle assembly. This ensures that, as the checkpoint begins to lose its inhibitory hold on the APC/C, the degradation of cyclin B1 does not drop below the level required to maintain CDK1 activity (Figure 6E).

As chromosomes align, and APC/C^{cdc20} activity begins to increase, an excess of cyclin B1 offers two advantages. First, free cyclin B1 may provide a decoy substrate for the APC/C, consistent with our finding that it is targeted in preference to CDK1-bound cyclin B1 while the checkpoint is still active (essentially acting as bait for the APC/C). Second, should any CDK1-bound cyclin B1 become an early target for degradation, sufficient surplus cyclin B1 is always available for CDK1 reactivation. This capacity to reactivate CDK1 is also likely critical because the relationship between cyclin B1 and CDK1 is dynamic. The consequence is a prolonged inhibition of anaphase and an extended prometaphase period that permits chromosome alignment and error correction. Therefore, though cyclin B1 destruction initiates in prometaphase, the loss of CDK1 activity initiates ~90 min later, closely coinciding with the time at which stable end-on kinetochore-microtubule attachments are formed and checkpoint proteins are maximally depleted from kinetochores (Kitajima et al., 2011; Lane et al., 2012). Our results show that, without sufficient free cyclin B1, the spindle checkpoint is unable to maintain prometaphase CDK1 activity for long enough to prevent division errors. Both the spindle checkpoint and an excess of cyclin B1 contribute to the low frequency of aneuploidies in mouse oocytes.

While our data demonstrate a requirement for excess cyclin B1, the presence of such a surplus potentially presents a

Figure 6. Restricting Excess Cyclin B1 Results in Division Errors in Oocytes

(A) Bar chart representing the outcome of MI divisions in control and cyclin B1 MO oocytes following live confocal imaging of oocytes expressing Map7-GFP (green) and SiR DNA (red). Oocytes were imaged every 15 min from 4 to 12 h post GVBD. Phenotype 1: DNA divides between the oocyte and a PB1 with normal morphology. Phenotype 2: DNA divides between the oocyte and a PB1 that is outside of the range of PB1 sizes in control oocytes from the same oocyte pool. Phenotype 3: the oocyte undergoes anaphase and cytokinesis; however, all DNA is extruded in 1 or more polar bodies. Phenotype 4: chromosomes appear to align; however, anaphase is prevented. Phenotype 5: anaphase occurs yet cytokinesis fails, and attempted PBs are reabsorbed. Phenotype 6: "cut" phenotype; cytokinesis takes place over unseparated chromosomes. Phenotype 7: DNA divides between the oocyte and a PB1 with normal morphology, yet chromosomes do not align prior to anaphase.

(B) Example images of spindle movements and division phenotypes represented in (A). Phenotypes are also available to view as movies in Video S1. Scale bar, 10 μm.

(C) Image representing spindle parameter measurements in live maturing control (n = 12) and cyclin B1 MO (n = 28) oocytes. As in Figures 5B and 5C, each parameter was measured relative to oocyte size. "Chromosome dispersion" = the distance over which chromosomes are dispersed over the inter-polar axis. "Distance from the cortex" = distance from the cortex to the nearest spindle pole in the direction of spindle movement.

(D) Graphs showing changes in oocyte spindle parameters (as indicated) between 5.5 h post GVBD and "pre-anaphase" (the last image collected before anaphase) in control (green, n = 12) and cyclin B1 MO oocytes (purple, n = 28). Gray lines highlight paired data measurements taken in the same oocyte. ** denotes a significant difference in means ($p < 0.005$). ↓ denotes a significant difference between the variance of the control dataset and the variance of the cyclin B1 MO dataset pre-anaphase. These data are also represented as boxplots in Figure S7. See Tables 1 and S1 for p values.

(E) Model of free cyclin B1 destruction ahead of CDK1-bound cyclin B1 destruction in MI mouse oocytes. As spindle checkpoint activity declines in late prometaphase, an excess of free cyclin B1 is targeted for destruction via its D-box (black) and PM motif (red) in preference to CDK1-bound cyclin B1. CDK1-bound cyclin B1 is initially preserved, targeted at a later time point via its D-box only, coinciding with more complete spindle checkpoint satisfaction and the establishment of stable end-on kinetochore attachments.

problem for the oocyte. Excessive amounts of APC/C substrate are known to block the cell cycle, presumably as the APC/C can be overwhelmed by many substrates in competition (Rape et al., 2006). Cdc20 may not be in large excess over substrates (Lu et al., 2015). Indeed, when the maintenance of the checkpoint is prevented in early prometaphase, the resultant D-box-mediated cyclin B1 degradation is slow (Figure 2B). The novel PM motif we have identified may also explain how oocytes can overcome this obstacle. The PM motif, only exposed when cyclin B1 is not bound to CDK1, not only provides an APC/C decoy but also progressively removes the excess of cyclin B1 that might otherwise later perturb MI exit. The metaphase drop in CDK1 activity (necessary to drive anaphase) can then be readily achieved by rapid D-box-mediated destruction of the smaller pool of active CDK1-bound cyclin B1. This is evident in our B1 MO "rescue" experiments: when we replaced excess endogenous cyclin B1 with WT-B1-V, we were able to rescue the B1 MO phenotype. However, where PMmut-B1-V was used in place of WT-B1-V, the majority of oocytes arrested in either metaphase or early anaphase (Figure 5D).

How the PM motif allows free cyclin B1 to bypass the checkpoint remains to be determined, though we would suggest that our results mimic some aspects of cyclin A destruction. Timely destruction of cyclin A in prometaphase is necessary for mitotic exit and requires sequences outside of the canonical D-box (Tin Su, 2001). The N terminus of cyclin A is able to bind Cdc20 with an affinity that may allow it to outcompete spindle checkpoint proteins (Di Fiore and Pines, 2010). However, the rate of cyclin A2 destruction may still be reduced by highly active checkpoint signaling (Boekhout and Wolthuis, 2015), presumably due to much greater competition for Cdc20 from checkpoint proteins. This suggests that the destruction of cyclin A2 remains at least partially coupled to the activity of the checkpoint in early prometaphase. Likewise, Y170A-B1 and n190-B1 (both representing free cyclin B1) show a reduced rate of destruction in a low concentration of nocodazole, which permits the assembly of a bipolar spindle but blocks the cell cycle, presumably due to impaired kinetochore-microtubule attachments (Wassmann et al., 2003). This could suggest that PM-motif-mediated destruction of free cyclin B1 might couple the timing of a prolonged prometaphase to the progressive attachment status of chromosomes in MI oocytes.

A major function of the ubiquitin-proteasome system is the disposal of misfolded proteins (Fredrickson et al., 2011; Rosenbaum et al., 2011). Similarly, subunits of protein complexes are often short lived if they do not become incorporated within their complex (Davey and Morgan, 2016; Harper and Bennett, 2016; Ravid and Hochstrasser, 2008). This suggests that in both situations, features that make proteins more susceptible to degradation are exposed in specific states. An excellent example of this is the *S. pombe* APC/C subunit Hcn1 and its partner Cut9/Apc6. Crystallography shows the acetylated "N-end rule" (Ac/N) degron of Hcn1 enclosed within Cut9 (Zhang et al., 2010), consistent with the proposal that masking of Ac/N degrons might control protein subunit stoichiometry (Hwang et al., 2010). Indeed, overexpression of Cut9 and "decoy" Hcn1 proteins provided support for this model (Shemorry et al., 2013). However, beyond such examples of protein quality control, there are few, if any, instances in which degron masking is used to regulate the activity of a key protein complex. The PM motif that we have identified clearly participates directly in the heterodimerization interface in the recently solved structure of cyclin B1 bound to CDK1 (Brown et al., 2015). Our results suggest that oocytes exploit an imbalance in protein subunit stoichiometry to maintain the activity of an essential cell cycle regulator over a timescale of hours. We demonstrate that overexpression of kinase-dead CDK1 protects cyclin B1, consistent with the idea that the imbalance in levels of cyclin B1 and CDK1 is coupled to a degron-masking mechanism, allowing the oocyte to overcome the unique set of challenges presented by MI spindle assembly. Synthesis of an excess of cyclin B1 containing a prometaphase degradation motif generates a decoy substrate, while the masking of this motif within the cyclin B1:CDK1 complex preserves essential CDK1 activity until chromosome alignment is complete. We suggest that degron-masking mechanisms may have more widespread functions than previously anticipated.

In the current manuscript, we have revised our understanding of the regulation of cyclin B1 and CDK1 activity in mouse oocytes. Our findings are important for understanding chromosome segregation errors in human oocytes. Aneuploidy is the number one genetic cause of miscarriage and birth defects in humans (Hassold and Hunt, 2001). Even in women under the age of 35, up to 30% of all zygotes are aneuploid, with 80–90% of the errors thought to originate in oocyte MI (Homer, 2011). It is likely that the balance of cyclin B1 and CDK1 in human oocytes contributes to embryo viability. Furthermore, given the general conservation of molecular mechanisms in the control of both mitotic and meiotic cell cycles, it is possible that the PM motif has additional mitotic functions in the "housekeeping" of cyclin B1 protein levels or the slippage of cells out of mitotic arrest. Beyond cell division, it is likely that the masking and unveiling of degrons has a key role to play in proteostasis.

STAR★METHODS

Detailed methods are provided in the online version of this paper and include the following:

- KEY RESOURCES TABLE
- CONTACT FOR REAGENT AND RESOURCE SHARING
- EXPERIMENTAL MODEL AND SUBJECT DETAILS
  - Gamete Collection and Culture
  - Mitotic Cell Cultures
- METHOD DETAILS
  - Preparation of cRNA Constructs for Microinjection
  - Drug Treatments of Oocytes
  - Knockdown of Gene Expression Using Morpholinos
  - Microinjection and Imaging
  - Molecular Structure Images
  - Overexpression of Reporter Constructs
  - Western Blotting
- QUANTIFICATION AND STATISTICAL ANALYSIS
  - Normalising Data in Individual Oocytes
  - Data Alignment within Treatment Groups
  - Data Alignment between Treatment Groups
  - Methods of Statistical Analysis

SUPPLEMENTAL INFORMATION

Supplemental Information includes seven figures, one table, and one video and can be found with this article online at https://doi.org/10.1016/j.devcel.2019.01.008.

ACKNOWLEDGMENTS

We thank M. Herbert and J.A. Endicott for critical reading of the manuscript and M. Sinclair and F. Davidson for technical assistance. This work was supported by a Wellcome Trust Career Re-entry Fellowship grant to S.M. (062376). J.M.G.H. is a Wellcome Trust Investigator (106951/Z/15/Z), was a Scholar of the Leukemia and Lymphoma Society (1028-12), and holds a Royal Society Wolfson Research Merit Award. O.R.D. is a Sir Henry Dale Fellow jointly funded by the Wellcome Trust and Royal Society (Grant Number 104158/Z/14/Z).

AUTHOR CONTRIBUTIONS

S.M. was responsible for the overall direction of the project. S.M. carried out most of the experiments alongside C.T. and M.D.L. S.M. and M.D.L. designed most of the experiments alongside J.M.G.H., and O.R.D., S.M., J.M.G.H., M.D.L., and C.T. prepared the manuscript.

DECLARATION OF INTERESTS

The authors declare no competing interests.

Received: May 17, 2018
Revised: October 22, 2018
Accepted: December 29, 2018
Published: February 7, 2019

REFERENCES

Arooz, T., Yam, C.H., Siu, W.Y., Lau, A., Li, K.K.W., and Poon, R.Y.C. (2000). On the concentrations of cyclins and cyclin-dependent kinases in extracts of cultured human cells. Biochemistry *39*, 9494–9501.

Bentley, A.M., Normand, G., Hoyt, J., and King, R.W. (2007). Distinct sequence elements of cyclin B1 promote localization to chromatin, centrosomes, and kinetochores during mitosis. Mol. Biol. Cell *18*, 4847–4858.

Boekhout, M., and Wolthuis, R. (2015). Nek2A destruction marks APC/C activation at the prophase-to-prometaphase transition by spindle-checkpoint-restricted Cdc20. J. Cell Sci. *128*, 1639–1653.

Brito, D.A., and Rieder, C.L. (2006). Mitotic checkpoint slippage in humans occurs via cyclin B destruction in the presence of an active checkpoint. Curr. Biol. *16*, 1194–1200.

Brown, N.R., Korolchuk, S., Martin, M.P., Stanley, W.A., Moukhametzianov, R., Noble, M.E.M., and Endicott, J.A. (2015). CDK1 structures reveal conserved and unique features of the essential cell cycle CDK. Nat. Commun. *6*, 6769.

Brunet, S., Maria, A.S., Guillaud, P., Dujardin, D., Kubiak, J.Z., and Maro, B. (1999). Kinetochore fibers are not involved in the formation of the first meiotic spindle in mouse oocytes, but control the exit from the first meiotic M phase. J. Cell Biol. *146*, 1–12.

Bulinski, J.C., Gruber, D., Faire, K., Prasad, P., and Chang, W. (1999). GFP chimeras of E-MAP-115 (ensconsin) domains mimic behavior of the endogenous protein in vitro and in vivo. Cell Struct. Funct. *24*, 313–320.

Clift, D., and Schuh, M. (2015). A three-step MTOC fragmentation mechanism facilitates bipolar spindle assembly in mouse oocytes. Nat. Commun. *6*, 7217.

Clute, P., and Pines, J. (1999). Temporal and spatial control of cyclin B1 destruction in metaphase. Nat. Cell Biol. *1*, 82–87.

Davey, N.E., and Morgan, D.O. (2016). Building a regulatory network with short linear sequence motifs: lessons from the degrons of the anaphase-promoting complex. Mol. Cell *64*, 12–23.

Davydenko, O., Schultz, R.M., and Lampson, M.A. (2013). Increased CDK1 activity determines the timing of kinetochore-microtubule attachments in meiosis I. J. Cell Biol. *202*, 221–229.

Di Fiore, B., and Pines, J. (2010). How cyclin A destruction escapes the spindle assembly checkpoint. J. Cell Biol. *190*, 501–509.

Fredrickson, E.K., Rosenbaum, J.C., Locke, M.N., Milac, T.I., and Gardner, R.G. (2011). Exposed hydrophobicity is a key determinant of nuclear quality control degradation. Mol. Biol. Cell *22*, 2384–2395.

Galli, M., and Morgan, D.O. (2016). Cell size determines the strength of the spindle assembly checkpoint during embryonic development. Dev. Cell *36*, 344–352.

Gavet, O., and Pines, J. (2010). Progressive activation of CyclinB1-Cdk1 coordinates entry to mitosis. Dev. Cell *18*, 533–543.

Glotzer, M., Murray, A.W., and Kirschner, M.W. (1991). Cyclin is degraded by the ubiquitin pathway. Nature *349*, 132–138.

Goda, T., Funakoshi, M., Suhara, H., Nishimoto, T., and Kobayashi, H. (2001). The N-terminal helix of Xenopus cyclins A and B contributes to binding specificity of the cyclin-CDK complex. J. Biol. Chem. *276*, 15415–15422.

Gorbsky, G.J. (2015). The spindle checkpoint and chromosome segregation in meiosis. FEBS J. *282*, 2471–2487.

Guharoy, M., Bhowmick, P., Sallam, M., and Tompa, P. (2016). Tripartite degrons confer diversity and specificity on regulated protein degradation in the ubiquitin-proteasome system. Nat. Commun. *7*, 10239.

Gui, L., and Homer, H. (2012). Spindle assembly checkpoint signalling is uncoupled from chromosomal position in mouse oocytes. Development *139*, 1941–1946.

Hagting, A., Den Elzen, N., Vodermaier, H.C., Waizenegger, I.C., Peters, J.M., and Pines, J. (2002). Human securin proteolysis is controlled by the spindle checkpoint and reveals when the APC/C switches from activation by Cdc20 to Cdh1. J. Cell Biol. *157*, 1125–1137.

Harper, J.W., and Bennett, E.J. (2016). Proteome complexity and the forces that drive proteome imbalance. Nature *537*, 328–338.

Hassold, T., and Hunt, P. (2001). To err (meiotically) is human: the genesis of human aneuploidy. Nat. Rev. Genet. *2*, 280–291.

He, J., Chao, W.C.H., Zhang, Z., Yang, J., Cronin, N., and Barford, D. (2013). Insights into degron recognition by APC/C coactivators from the structure of an Acm1-Cdh1 complex. Mol. Cell *50*, 649–660.

Herbert, M., Levasseur, M., Homer, H., Yallop, K., Murdoch, A., and Mcdougall, A. (2003). Homologue disjunction in mouse oocytes requires proteolysis of securin and cyclin B1. Nat. Cell Biol. *5*, 1023–1025.

Holubcová, Z., Blayney, M., Elder, K., and Schuh, M. (2015). Human oocytes. Error-prone chromosome-mediated spindle assembly favors chromosome segregation defects in human oocytes. Science *348*, 1143–1147.

Homer, H. (2011). New insights into the genetic regulation of homologue disjunction in mammalian oocytes. Cytogenet. Genome Res. *133*, 209–222.

Homer, H.A., Mcdougall, A., Levasseur, M., Yallop, K., Murdoch, A.P., and Herbert, M. (2005). Mad2 prevents aneuploidy and premature proteolysis of cyclin B and securin during meiosis I in mouse oocytes. Genes Dev. *19*, 202–207.

Hwang, C.S., Shemorry, A., and Varshavsky, A. (2010). N-terminal acetylation of cellular proteins creates specific degradation signals. Science *327*, 973–977.

Izawa, D., and Pines, J. (2011). How APC/C-Cdc20 changes its substrate specificity in mitosis. Nat. Cell Biol. *13*, 223–233.

Kitajima, T.S., Ohsugi, M., and Ellenberg, J. (2011). Complete kinetochore tracking reveals error-prone homologous chromosome biorientation in mammalian oocytes. Cell *146*, 568–581.

Kolano, A., Brunet, S., Silk, A.D., Cleveland, D.W., and Verlhac, M.H. (2012). Error-prone mammalian female meiosis from silencing the spindle assembly checkpoint without normal interkinetochore tension. Proc. Natl. Acad. Sci. U S A *109*, E1858–E1867.

Lane, S.I.R., and Jones, K.T. (2014). Non-canonical function of spindle assembly checkpoint proteins after APC activation reduces aneuploidy in mouse oocytes. Nat. Commun. *5*, 3444.

Lane, S.I.R., Yun, Y., and Jones, K.T. (2012). Timing of anaphase-promoting complex activation in mouse oocytes is predicted by microtubule-kinetochore attachment but not by bivalent alignment or tension. Development *139*, 1947–1955.

Lara-Gonzalez, P., Westhorpe, F.G., and Taylor, S.S. (2012). The spindle assembly checkpoint. Curr. Biol. *22*, R966–R980.

Ledan, E., Polanski, Z., Terret, M.E., and Maro, B. (2001). Meiotic maturation of the mouse oocyte requires an equilibrium between cyclin B synthesis and degradation. Dev. Biol. *232*, 400–413.

Lemaire, P., Garrett, N., and Gurdon, J.B. (1995). Expression cloning of Siamois, a Xenopus homeobox gene expressed in dorsal-vegetal cells of blastulae and able to induce a complete secondary axis. Cell *81*, 85–94.

Leroy, D., Birck, C., Brambilla, P., Samama, J.P., and Ducommun, B. (1996). Characterisation of human cdc2 lysine 33 mutations expressed in the fission yeast Schizosaccharomyces pombe. FEBS Lett. *379*, 217–221.

Levasseur, M. (2013). Making cRNA for microinjection and expression of fluorescently tagged proteins for live-cell imaging in oocytes. In Mammalian Oocyte Regulation: Methods and Protocols, H.A. Homer, ed. (Humana Press), pp. 121–134.

Levasseur, M., Dumollard, R., Chambon, J.P., Hebras, C., Sinclair, M., Whitaker, M., and Mcdougall, A. (2013). Release from meiotic arrest in ascidian eggs requires the activity of two phosphatases but not CaMKII. Development *140*, 4583–4593.

Lu, D., Girard, J.R., Li, W., Mizrak, A., and Morgan, D.O. (2015). Quantitative framework for ordered degradation of APC/C substrates. BMC Biol. *13*, 96.

Madgwick, S., Hansen, D.V., Levasseur, M., Jackson, P.K., and Jones, K.T. (2006). Mouse Emi2 is required to enter meiosis II by reestablishing cyclin B1 during interkinesis. J. Cell Biol. *174*, 791–801.

Madgwick, S., Nixon, V.L., Chang, H.Y., Herbert, M., Levasseur, M., and Jones, K.T. (2004). Maintenance of sister chromatid attachment in mouse eggs through maturation-promoting factor activity. Dev. Biol. *275*, 68–81.

Nagaoka, S.I., Hodges, C.A., Albertini, D.F., and Hunt, P.A. (2011). Oocyte-specific differences in cell-cycle control create an innate susceptibility to meiotic errors. Curr. Biol. *21*, 651–657.

Nixon, V.L., Levasseur, M., Mcdougall, A., and Jones, K.T. (2002). Ca(2+) oscillations promote APC/C-dependent cyclin B1 degradation during metaphase arrest and completion of meiosis in fertilizing mouse eggs. Curr. Biol. *12*, 746–750.

Pan, H., Ma, P., Zhu, W., and Schultz, R.M. (2008). Age-associated increase in aneuploidy and changes in gene expression in mouse eggs. Dev. Biol. *316*, 397–407.

Pines, J. (2011). Cubism and the cell cycle: the many faces of the APC/C. Nat. Rev. Mol. Cell Biol. *12*, 427–438.

Prodon, F., Chenevert, J., Hébras, C., Dumollard, R., Faure, E., Gonzalez-Garcia, J., Nishida, H., Sardet, C., and Mcdougall, A. (2010). Dual mechanism controls asymmetric spindle position in ascidian germ cell precursors. Development *137*, 2011–2021.

Rape, M., Reddy, S.K., and Kirschner, M.W. (2006). The processivity of multiubiquitination by the APC determines the order of substrate degradation. Cell *124*, 89–103.

Ravid, T., and Hochstrasser, M. (2008). Diversity of degradation signals in the ubiquitin-proteasome system. Nat. Rev. Mol. Cell Biol. *9*, 679–690.

Reis, A., Madgwick, S., Chang, H.Y., Nabti, I., Levasseur, M., and Jones, K.T. (2007). Prometaphase APCcdh1 activity prevents non-disjunction in mammalian oocytes. Nat. Cell Biol. *9*, 1192–1198.

Rosenbaum, J.C., Fredrickson, E.K., Oeser, M.L., Garrett-Engele, C.M., Locke, M.N., Richardson, L.A., Nelson, Z.W., Hetrick, E.D., Milac, T.I., Gottschling, D.E., et al. (2011). Disorder targets misorder in nuclear quality control degradation: a disordered ubiquitin ligase directly recognizes its misfolded substrates. Mol. Cell *41*, 93–106.

Santamaría, D., Barrière, C., Cerqueira, A., Hunt, S., Tardy, C., Newton, K., Cáceres, J.F., Dubus, P., Malumbres, M., and Barbacid, M. (2007). Cdk1 is sufficient to drive the mammalian cell cycle. Nature *448*, 811–815.

Sebestova, J., Danylevska, A., Novakova, L., Kubelka, M., and Anger, M. (2012). Lack of response to unaligned chromosomes in mammalian female gametes. Cell Cycle *11*, 3011–3018.

Shemorry, A., Hwang, C.S., and Varshavsky, A. (2013). Control of protein quality and stoichiometries by N-terminal acetylation and the N-end rule pathway. Mol. Cell *50*, 540–551.

Solc, P., Kitajima, T.S., Yoshida, S., Brzakova, A., Kaido, M., Baran, V., Mayer, A., Samalova, P., Motlik, J., and Ellenberg, J. (2015). Multiple requirements of PLK1 during mouse oocyte maturation. PLoS One *10*, e0116783.

Sullivan, M., and Morgan, D.O. (2007). Finishing mitosis, one step at a time. Nat. Rev. Mol. Cell Biol. *8*, 894–903.

Tin Su, T. (2001). Cell cycle: how, when and why cells get rid of cyclin A. Curr. Biol. *11*, R467–R469.

Todaro, G.J., and Green, H. (1963). Quantitative studies of the growth of mouse embryo cells in culture and their development into established lines. J. Cell Biol. *17*, 299–313.

Varshavsky, A. (1991). Naming a targeting signal. Cell *64*, 13–15.

Verlhac, M.H., Lefebvre, C., Guillaud, P., Rassinier, P., and Maro, B. (2000). Asymmetric division in mouse oocytes: with or without Mos. Curr. Biol. *10*, 1303–1306.

Wassmann, K., Niault, T., and Maro, B. (2003). Metaphase I arrest upon activation of the Mad2-dependent spindle checkpoint in mouse oocytes. Curr. Biol. *13*, 1596–1608.

Wolthuis, R., Clay-Farrace, L., Van Zon, W., Yekezare, M., Koop, L., Ogink, J., Medema, R., and Pines, J. (2008). Cdc20 and Cks direct the spindle checkpoint-independent destruction of cyclin A. Mol. Cell *30*, 290–302.

Yamano, H., Tsurumi, C., Gannon, J., and Hunt, T. (1998). The role of the destruction box and its neighbouring lysine residues in cyclin B for anaphase ubiquitin-dependent proteolysis in fission yeast: defining the D-box receptor. EMBO J. *17*, 5670–5678.

Zhang, Z., Kulkarni, K., Hanrahan, S.J., Thompson, A.J., and Barford, D. (2010). The APC/C subunit Cdc16/Cut9 is a contiguous tetratricopeptide repeat superhelix with a homo-dimer interface similar to Cdc27. EMBO J. *29*, 3733–3744.

van Zon, W.V., and Wolthuis, R.M.F. (2010). Cyclin A and Nek2A: APC/C–Cdc20 substrates invisible to the mitotic spindle checkpoint. Biochem. Soc. Trans. *38*, 72–77.

STAR★METHODS

KEY RESOURCES TABLE

REAGENT OR RESOURCE	SOURCE	IDENTIFIER
Antibodies		
Anti-cyclin B1	Abcam	Cat# ab72 [V152] RRID:AB_305751
Anti-CDK1/CDK2	Santa Cruz	Cat# sc-53219 [AN21.2] RRID:AB_2120095
Anti–mouse IgG	Cell Signaling Technology	Cat# 7076P2, RRID:AB_330924
Bacterial and Virus Strains		
Cdk1 FRET Sensor (2372)	Addgene (Gavet and Pines, 2010)	RRID:Addgene_26064
Inactive Cdk1 FRET Sensor (2328)	Gift from Jonathan Pines (Gavet and Pines, 2010)	RRID:Addgene_26065
K33ACDK1	Gift from Jonathan Pines	N/A
Unmodified pRN3 vector	A gift from Patrick Lemaire (Lemaire et al., 1995)	N/A
Modified pRN3 vector	Levasseur, 2013	N/A
Chemicals, Peptides, and Recombinant Proteins		
M2 medium	Sigma	Cat# M7167 MDL# MFCD00283761
3-isobutyl-1-methylxanthine	Sigma	Cat# I5879 CAS# 28822-58-4
Nocodazole	Sigma	Cat# M1404 CAS# 31430-18-9
Flavopiridol	Santa Cruz	Cat# sc-202157 CAS# 146426-40-6
Reversine	Sigma	Cat# R3904 Cas# 656820-32-5
Cycloheximide	Sigma	Cat# 01810 Cas# 66-81-9
Hoechst 33342	Sigma	Cat# 14533 Cas# 23491-52-3
SiR-DNA	Spirochrome	Cat# CHF280.00
DMEM media	Lonza	Cat# 12-604
Fetal Bovine Serum	Gibco	Cat# 16000044
Recombinant CDK1 protein	A gift from Jane Endicott (Brown et al., 2015)	N/A
CDK1/Cyclin B Recombinant Human Protein	Thermo Fisher	PV3292
Critical Commercial Assays		
T3 mMESSAGE mMACHINE	Ambion	Cat# AM1348
GeneEditor	Promega	Cat# Q9280
ECL Select detection reagents	GE Healthcare	Cat# RPN2235
Experimental Models: Cell Lines		
U2OS cells	A gift from Neil Perkins	(RRID:CVCL_0042)
MEF cells	A gift from Neil Perkins	N/A
Experimental Models: Organisms/Strains		
Oocytes from 4-8 week old CD1 mice	Charles River	IMSR Cat# CRL:22, RRID: IMSR_CRL:22
Oligonucleotides		
Human cyclin B1 primers (Genbank: NM_031966)	Life Technologies	N/A
Human securin primers (Genbank: AJ223953.1)	Life Technologies	N/A

(*Continued on next page*)

Continued		
REAGENT OR RESOURCE	SOURCE	IDENTIFIER
Human K33ACDK1 primers (UGID:5796252)	Life Technologies	N/A
APC3 MO<br>CTTGAGGCTCAGACCCACTTTCTGC	Gene Tools	N/A
APC3 MO 5-base-mismatch<br>CTT**C**A**C**GCT**G**AGACCCACTTT**G**T**C**C	Gene Tools	N/A
Cdc20 MO<br>CGCTCTCGAACACGAACTGCGCCAT	Gene Tools	N/A
Cyclin B1 MO<br>TGTTCCTAGTGACCCTGAGCGCCAT	Gene Tools	N/A
Cyclin B1 MO 5-base-mismatch<br>T**C**TTCCTA**C**TGA**A**CCTGA**C**CGCA**A**T	Gene Tools	N/A
Software and Algorithms		
Metafluor 7.7.0.0	Molecular Devices	RRID:SCR_014294
NIS Elements AR version 5.02.01	Nikon	RRID:SCR_014329
Image J	Image J	RRID:SCR_003070
SPSS	IBM	RRID:SCR_002865
PyMOL Molecular Graphics System Version 1.3	Schrödinger, LLC	RRID:SCR_000305
Other		
Inverted epifluorescence microscope	Olympus	Cat# 1X71
Confocal laser microscope	Nikon	Cat# A1R

CONTACT FOR REAGENT AND RESOURCE SHARING

Further information and requests for resources and reagents should be directed to and will be fulfilled by the Lead Contact, Suzanne Madgwick (suzanne.madgwick@newcastle.ac.uk).

EXPERIMENTAL MODEL AND SUBJECT DETAILS

Gamete Collection and Culture

4 to 8-week-old female, outbred, CD1 mice (Charles River; RRID:IMSR_CRL:22) were used. All animals were handled in accordance with ethics approved by the UK Home Office Animals (Scientific Procedures) Act 1986. GV oocytes were collected from ovaries punctured with a sterile needle; oocytes were stripped of their cumulus cells mechanically using a pipette. For bench handling, microinjections, and imaging experiments, oocytes were cultured at 37°C in medium M2 (Sigma), with the addition of 30 nM 3-isobutyl-1-methylxanthine (IBMX; Sigma) to arrest oocytes at prophase I where necessary. Data was only collected from oocytes which underwent GVBD with normal timings and had a diameter within 95-105% of the population average. To ensure reproducibility, oocyte data sets were gathered from a minimum of 3 independent experiments. For each independent experiment both control and treatments groups were derived from the same pool of oocytes collected from a minimum of 2 animals. Oocytes were selected at random for microinjection, however the investigators were not blinded to allocation during experiments or outcome assessment.

Mitotic Cell Cultures

U2OS cells (an immortalised cell line from a female patient with osteosarcoma; RRID:CVCL_0042) and Mouse Embryonic Fibroblasts (MEFs) were used as western blotting standards and were a gift from Neil Perkins. The sex of the embryonic mice for the MEF cells was not determined as this was not procedure at the time. U2OS cells were maintained at 37°C, 5% CO2 in DMEM (Lonza) with 10% FBS (Life Technologies) and antibiotics. While U2OS cells are a standard laboratory cell line, MEFs were isolated as follows; internal torso connective tissue from 13.5-day embryos was washed in sterile PBS and minced in 1x Trypsin (Invitrogen) for 15 min at 37°C. The sex of the embryonic mice was not determined. Following repeated pipetting to break up large tissue fragments, the cell pellet was resuspended in DMEM (Lonza) supplemented with 20% Fetal Bovine Serum (FBS) (Gibco, Paisley, UK) and 50U/ml penicillin/streptomycin (Lonza), and incubated at 37°C in a 5% CO_2 humidified atmosphere. Once cells reached 90% confluency, they were sub-cultured in 75cm^2 flasks and considered as passage 1. Cells were then cultured following the standard 3T3 protocol (Todaro and Green, 1963). Cells were considered immortalised beyond passage 14, but not used in experiments beyond passage 25.

METHOD DETAILS

Preparation of cRNA Constructs for Microinjection

The Y170A mutant of cyclin B1 was made using the GeneEditor (Promega) *in vitro* mutagenesis system according to the manufacturer's protocol. Microtubules were visualised by expression of mRNA coding for the microtubule binding protein Map7 fused to GFP (Prodon et al., 2010). Wild type human cyclin B1 (NM_031966), human securin (AF095287.1) and human CDK1 K33A (a gift from Jonathon Pines) sequences were amplified by PCR as previously described (Madgwick et al., 2004). Further cyclin B1 mutations were generated by primer overhang extension PCR. Δ90 cyclin B1 was cloned into a pRN3 vector (a gift from Patrick Lamaire; (Lemaire et al., 1995) while all other amplified sequences were cloned into a modified pRN3 vector designed to produce mRNA transcripts C-terminally coupled to Venus or Cerulean fluorescent proteins (Levasseur, 2013). The CDK1 FRET sensor (Addgene plasmid #26064) and inactive sensor (a gift from Jonathon Pines) sequences were amplified by PCR and cloned into the pRN3 vector. Maximal stability was conferred on all cRNA constructs by the presence of a 5′ globin UTR upstream and both 3′UTR and poly (A)–encoding tracts downstream of the gene. cRNA for microinjection was synthesized using T3 mMESSAGE mMACHINE (Ambion) and dissolved in nuclease-free water to the required micropipette concentration.

Drug Treatments of Oocytes

At the times indicated, nocodazole (Sigma) was added to media at a concentration of either 100 or 500 nM, the CDK1 inhibitor flavopiridol (Santa Cruz) at 1 μM (Solc et al., 2015), the Mps1 inhibitor reversine (Sigma) at 100 nM (Kolano et al., 2012) and the protein synthesis inhibitor cycloheximide (Sigma) at 10 μg/ml (Madgwick et al., 2006). Where used, Hoechst 33342 at 10 μg/ml (Sigma) was added to media for 15 min prior to imaging. For longer term confocal time-lapse imaging, DNA was stained with SiR-DNA at 250 nM (Spirochrome).

Knockdown of Gene Expression Using Morpholinos

Morpholino antisense oligos (MO; Genetools) designed to recognize the 5′UTR of cyclin B1, Cdc20 and APC3 were microinjected at a micropipette concentration of 1 mM. Cyclin B1 MO and cyclin B1 5 base pair mismatch (5bpmm) MO oocytes were released from arrest within 30 minutes of injection since we did not want to perturb prophase levels of cyclin B1, but only to prevent such excessive accumulation in prometaphase. APC3 MO and APC3 5bpmm MO injected oocytes were arrested in prophase with IBMX for 6 hours prior to release. A short Cdc20 MO incubation period (1.5-2 hours) was carried out as previously described (Reis et al., 2007). A longer Cdc20 MO incubation period required the use of cycloheximide since oocytes began to escape IBMX induced prophase arrest approximately 3 hours after injection of the Cdc20 MO. We reasoned that, without sufficient Cdc20 available to suppress prometaphase cyclin B1 levels, a premature increase in cyclin B1 drove oocytes out of prophase. Therefore, a longer Cdc20 MO incubation period included cycloheximide to prevent this. To release prophase arrest, both IBMX and cycloheximide were washed out from oocyte containing media; the use of cycloheximide in prophase arrest did not perturb subsequent MI maturation or cyclin B1 destruction profiles in control oocytes.

Microinjection and Imaging

Oocyte microinjection of MOs and cRNA constructs was carried out on the heated stage of an inverted epifluorescence microscope (Olympus; 1X71). In brief, fabricated micropipettes were inserted into cells using the negative capacitance overcompensation facility on an electrophysiological amplifier (World Precision Instruments); this procedure ensures a high rate of survival (>95%). For destruction profiles, images were captured on an Olympus IX71 inverted epifluorescence microscope using a CCD camera (Micromax, Sony Interline chip, Princeton Instruments), analysed and processed using MetaFluor software (version 7.7.0.0; Molecular Devices). All experiments were performed at 37°C. To generate fluorescent protein profiles, bright-field and fluorescence images were recorded at 10 minute intervals. Confocal images were collected on a Nikon A1R confocal laser microscope. Imaging began ~4 hours post GVBD to minimise oocyte laser exposure. Oocytes were imaged at 15 minute intervals through 15+ Z-sections over an 8-hour period in a temperature-controlled, humidified chamber at 37°C. Fluorescent images were recorded and processed in NIS-Elements (Nikon).

Molecular Structure Images

Molecular structure images were generated using the PyMOL Molecular Graphics System, Version 1.3 Schrödinger, LLC.

Overexpression of Reporter Constructs

The maximum level of expression of all non-CDK1 binding cyclin B1 reporter constructs was kept low (~8-13% of endogenous; Figure S1G) and did not perturb meiosis I progression as determined by identical mean GVBD and PB1 extrusion timings (compared to uninjected oocytes from the same pool), and by recording the timing of the establishment of a barrel shaped spindle by time lapse microscopy. Hence, where oocytes are aligned at PB1, the mean GVBD to PB1 timings between groups are always identical. All comparisons only include data from oocytes expressing cyclin B1 constructs to similar levels (see Figure S3A for example). Given the 1:1 ratio of the cyclin B1 to FP component of each construct, we reasoned that comparable fluorescence levels equate to comparable molar amounts. Oocytes with either excessive starting amounts of reporter proteins, or with excessive translation rates were discounted. Where cyclin B1 constructs were not used to report destruction timing; Δ90 cyclin B1 in Figure S5, and WT-B1-V in MO 'rescue' experiments (Figure 5D); cRNA was injected to generate expression at ~80% endogenous relative to a control oocyte.

Western Blotting

Mitotic cells were prepared by lysis in Laemmli buffer following mechanical shake off of mitotic cells after 8 h incubation in 100 nM nocodazole. Oocytes were collected 5.5 hours after GVBD ± 15 min and lysed in Laemmli buffer. The cyclin B1:CDK1 purified complex was purchased from Thermo Fisher Scientific; PV3292. SDS-PAGE and immunoblotting was conducted by standard procedures. For cyclin B1, immunoblots were incubated for 2 hours with anti-cyclin B1 (Abcam ab72) at 1:250. For CDK1, immunoblots were incubated for 2 hours with anti-CDK1/CDK2 (Santa Cruz sc-53219) at 1:200. Non-fat milk (5%) was used as a blocking solution. Anti–mouse IgG (7076P2; Cell Signaling) and ECL Select (RPN2235; GE Healthcare) were used as secondary detection reagents. ECL Select detection reagents were specifically used to produce x-ray signals with broad linear dynamic range. The intensity of each band was then determined in Image-J after scanning x-ray film. Band intensities were plotted against oocyte numbers in Excel. The equation of the line generated by these points was used to determine the relative amount in cyclin B1 MO oocytes. Note in Figure S4F, the band intensity of the cyclin B1 MO oocyte lane is within our standard curve. Immunoblots S1A + D are representative of 3 independent blots. Immunoblot S1F is representative of 2 independent blots.

QUANTIFICATION AND STATISTICAL ANALYSIS

All relevant data are available from the authors. Where fluorescence values are used to generate destruction profiles, each curve represents the mean set of values recorded from a number of oocytes (n = stated in figure legends). As stated within figure legends, error bars = SEM throughout. The extent of the effect of mutating and truncating cyclin B1 on subsequent destruction timings was not pre-specified therefore no statistical method was used to predetermine sample size.

Normalising Data in Individual Oocytes

In the majority of experiments, where fluorescent proteins displayed a period of destruction, the peak fluorescence value prior to destruction was normalised to 100 au. Where a treatment did not result in a period of destruction (Figures 2A, 2C, 2D, 3D, and 4C), values were instead normalised to 50 au at GVBD (reflecting the fact that cyclin B1 construct fluorescence typically doubled in intensity from GVBD to its maximum). Similarly, where the CDK1 FRET sensor showed a loss of FRET signal, the peak signal prior to the drop was normalised to 100 au. On two occasions treatment did not result in a loss of FRET signal (Figure S5; Δ90 cyclin B1 and inactive FRET sensor traces). Δ90 cyclin B1 values were instead normalised to 90 au at GVBD reflecting the fact that the FRET signal typically increased by 10% from GVDB to its maximum. Inactive FRET values were instead normalised to 75 au at GVBD reflecting the fact that the inactive FRET ratio was typically 75% of the maximum active FRET signal in late prometaphase oocytes.

Data was normalised in order to give each fluorescence profile the same importance regardless of starting or maximum fluorescence value. However, like examples in Figures S3A, S3B, S6A, and S6B, we also compared mean traces generated by raw data (aligned to both GVBD and PB1). As expected, in each case, while raw data figures are naturally more variable at individual time points, the order of construct destruction remains the same.

Data Alignment within Treatment Groups

Where the focus of the experiment was to determine the length of the destruction period prior PB1 extrusion, fluorescence curves from individual oocytes were aligned at the point of PB1 extrusion. Where determining the effect of a drug was the focus of the experiment, fluorescence curves were aligned at the point of drug addition. Where treatment did not result in either a loss of fluorescence or PB1 extrusion, fluorescence traces were aligned at GVBD. Means for each treatment group at each time point were then calculated. Error bars equal S.E.M throughout.

Data Alignment between Treatment Groups

For comparison, where the fluorescence traces from different types of treatment are shown alongside each other (Figures 2A, 2C, 2D, 4C, 5A, 5B, and S5) mean fluorescence curves are aligned by matching mean GVBD time points.

Methods of Statistical Analysis

In Figure 4D destruction rates were expressed as $T_{1/2}$ values in minutes, calculated using the formula $T_{1/2}=\ln(0.5)/k$, where $k=-1/T \times \ln(t_1/t_2)$, where T is the time interval between t_1 and t_2, and where t_1 and t_2 are the start and finish fluorescence values (Levasseur et al., 2013). Statistical comparison of destruction rates of mean $T_{1/2}$ values was by two-sample two-tailed t-test; a null hypothesis was rejected if $P<0.05$.

Spindle parameter measurements were recorded in NIS-Elements (Nikon) after confocal imagining. To determine the most appropriate statistical test for data analysis, data sets were first checked for distribution type. By ‘Shapiro-Wilk’ testing, spindle lengths, spindle widths and chromosome dispersion distances were judged to be normally distributed. Spindle distances from the cortex and PB1 sizes were not judged to be normally distributed. All tests were performed in SPSS.

Table 1; for non-paired data sets where data sets were judged to be normally distributed, ‘Levene’s Test’ was carried out to judged equality of variances. Once it was determined whether equal variance could or could not be assumed, the relevant ‘Independent

Samples Test' 2-tailed P-value determined the significance of differences between means. For non-paired data sets where data sets were not judged to be normally distributed (PB1 size), non-parametric 'Mann-Whitney U Tests' were used to determine the significance of differences between means.

Table S1; for normally distributed paired data sets, paired sample t-tests were used to determine the significance of differences in means between time points. For paired data sets not judged to be normally distributed, Wilcoxon Signed Ranks Tests were used to determine the significance of differences in means between time points.

Differences in PB1 sizes between groups. Figure 5D data sets are non-paired and not judged to normally distributed, therefore non-parametric 'Mann-Whitney U Tests' were used to determine the significance of differences in means.

Developmental Cell, Volume *48*

Supplemental Information

Aneuploidy in Oocytes Is Prevented by Sustained

CDK1 Activity through Degron Masking in Cyclin B1

Mark D. Levasseur, Christopher Thomas, Owen R. Davies, Jonathan M.G. Higgins, and Suzanne Madgwick

Supplementary Figure and Table Legends

Figure S1. Related to Fig. 1. Representative raw data destruction profiles of D-box-B1-C (cerulean, pink) and Y170A-B1-V (blue) co-expressed in the same oocyte. (A) in an untreated oocyte, and **(B)** in oocytes on addition of cycloheximide as indicated (CHX)**.** Note that the order of D-box-B1 and Y170A-B1 destruction did not depend on continued protein synthesis (which is prevented by cycloheximide), nor on their relative expression levels (**i + ii** comparison). PB1 extrusion time as indicated.

Figure S2. Further destruction profiles of cyclin B1 truncations and mutations, an extension of Fig. 3. (A) NTH sequence detail and nomenclature of venus tagged cyclin B1 mutants. **(B)** Mean destruction profiles of D-box-B1+NTH (n=22), D-box-B1+NTH DIY-A (n=18) and D-box-B1-V (n=34). **(C)** Mean destruction profiles of n190Y170A-B1 (n=36), n190LRQL-A (n=13), and n190DIY-A (n=21). Cyclin B1 mutants included in parts 'C' carry an additional I150T mutation. This residue falls within cyclin B1's nuclear export signal, a sequence thought to be redundant in oocytes which do not have a nucleus. **(D)** Destruction profiles of Y170A-B1 and PMmut-B1 with (Y170A-B1* and PMmut-B1*) and without (Y170A-B1 and PMmut-B1) an I150T point mutation demonstrating that this amino acid switch did not affect either the timing, or the extent of destruction. Error bars ± SEM throughout.

Figure S3. Related to Fig. 3C. Examples of individual cyclin B1 destruction traces. (A) Raw data demonstrating protein expression ranges are similar for PMmut-B1-V and Y170A-B1-V. Given that the cyclin B1 and fluorescent component of each construct are physically linked, we reasoned that by matching fluorescence levels, different cyclin B1 reporters are expressed at similar levels. Y170A-B1-V expressing oocytes and PMmut-B1-V expressing oocytes are aligned to PB1 extrusion within treatment groups. Mean PB1 extrusion timings between treatment groups are identical. Thin traces represent individual oocytes while heavy traces indicate the mean. **(B)** Raw data above processed to normalize fluorescence values to 100%, 2.5 hours ahead of PB1 extrusion. This time point was selected as the initiation of cyclin B1 destruction in the average oocyte. Note that normalising data does not affect the observed order of destruction. Thin traces represent individual oocytes, while heavy traces indicate the mean. **(C)** Mean destruction profiles of Y170A-B1-V (n=18) and PMmut-B1-V (n=18) after addition of cycloheximide (CHX) at the time indicated. Individual traces were aligned to GVBD and normalised to 100 a.u. at the point of CHX addition. Error bars ± SEM. **(D)** Oocytes 5.5hrs post GVBD expressing Y170A-B1-V and PMmut-B1-V, incubated in the DNA dye SiR-DNA.

Figure S4. Related to Fig. 4. Quantification of cyclin B1 and CDK1 in mouse oocytes 5.5 h post GVBD using a 1:1 cyclin B1:CDK1 protein complex. (A) Western blot of known amounts of cyclin B1 and CDK1 recombinant protein (purified complex) alongside known numbers of mitotic U2OS cells to quantify cyclin B1 and CDK1 protein bands in U2OS cells (cell numbers indicated above). **(B)** Band densities of 'purified complex' lanes in part 'A' plotted relative to protein amount in ng. **(C)** Calculated amounts of cyclin B1 and CDK1 in 1000 U2OS cells from 'A' using the equation from 'B'. **(D)** Western blot of mitotic mouse embryonic fibroblasts (MEFs), U2OS cells, oocytes collected 5.5 hours post GVBD and 1.5 ng of cyclin B1 + 1.5 ng of CDK1 recombinant protein (cell numbers indicated above). Note the difference in the balance of cyclin B1 and CDK1 in the mitotic cell cycles of MEF's where CDK1 is in excess. **(E)** Using the same strategy as in 'B' and 'C', cyclin B1 and CDK1

band densities from part 'D' were used to relate cyclin B1 and CDK1 protein levels in oocytes to an equivalent number of U2OS cells. From this cyclin B1 and CDK1 protein amounts were calculated per oocyte. We calculate the ratio of cyclin B1:CDK1 to be approximately 6:1. All mitotic cell lysates are prepared from cells arrested in nocodazole and collected by shake off.

(F) Western blots of control and cyclin B1 morpholino (MO) injected oocytes collected 5.5 hours post GVBD (numbers of oocytes loaded per lane are indicated). The upper blot was probed for cyclin B1, and the lower for CDK1 (2 different exposures). Quantification of protein bands indicates a 67% knockdown of cyclin B1 in cyclin B1 morpholino injected oocytes. The loss of cyclin B1 also results in a 12% loss of CDK1 protein. The ratio of cyclin B1:CDK1 was therefore calculated to be ~2.25:1 in cyclin B1 MO oocytes 5.5 hours post GVBD. Note that CDK1 levels are unchanged between GV stage and 5.5 hours post GVBD. **(G)** Western blot of 40 oocytes injected with Y170A-B1-V, probed for cyclin B1. Oocytes were collected and lysed 5 hours post GVBD. In order to be sure of detecting exogenous cyclin B1, 4-fold greater needle concentrations of cRNA were used for microinjection of blotting oocytes than those used to report cyclin B1 destruction profiles. We determined that all cyclin B1 reporters are expressed at between 8-13% of endogenous at their peak prior to destruction. All western blot images presented are scanned X-ray film.

Figure S5. Related to Fig. 4B. Validation of the CDK1 activity FRET sensor in oocytes. FRET ratios from oocytes expressing a CDK1 activity biosensor are able to detect changes in the balance between cyclin B1:CDK1 kinase activity and antagonistic phosphatase(s) activity. **(A)** Mean FRET ratios in oocytes after: **green trace,** no further treatment (n=72); **blue trace,** addition of the CDK1 inhibitor flavopiridol, demonstrating a rapid reduction in FRET signal (n=12); **yellow trace,** following overexpression of non-destructible cyclin B1 (Δ90 cyclin B1 lacks a D-box, n=23); **red trace,** inactive control biosensor in which the phosphorylated Ser126 is mutated to Ala (n=12). Inhibitors were added to the medium and PBs were extruded at the times indicated.

The Δ90 cyclin B1 experiment demonstrates that the pre-anaphase loss of FRET signal in control oocytes is the result of CDK1-bound cyclin B1 destruction. Oocytes overexpressing Δ90 cyclin B1 show no loss of FRET, indicating a stable balance between CDK1 kinase activity and phosphatase activity. In control oocytes, the loss of CDK1 activity switches the balance in favour phosphatase activity and the FRET ratio drops. This experiment also demonstrates that CDK1 levels, not cyclin B1 levels, are rate limiting since CDK1 activity in oocytes overexpressing Δ90 cyclin B1 does not increase beyond that of control cells.

(B) Mean FRET ratios from a pool of oocytes expressing the CDK1 biosensor following either no further treatment (**green trace** n=18) or microinjection of 2pg of unlabelled recombinant CDK1 protein, (**black trace** n=17/29). Oocytes injected with CDK1 are delayed in PB1 extrusion as indicated, presumably due to the need to disassemble greater numbers of cyclin B1:CDK1 complexes. 12/29 CDK1 protein injected oocytes failed to extrude a polar body and are not included. Given that the loss of CDK1 activity must be abrupt to generate an irreversible anaphase switch (Holt et al., 2008), we reasoned that the time lag in destroying more CDK1 associated cyclin B1 perturbed this switch resulting in a stalled anaphase. This experiment serves to demonstrate that we are working within the dynamic range of the FRET sensor and provides further evidence that CDK1 is rate limiting. Means ± SEM are shown.

Figure S6. Related to Fig 5. D-box substrates are targeted earlier where the amount of excess cyclin B1 is restricted. (A) Example replicate showing mean, raw data, CDK1 activity FRET ratios in untreated (n=18) and cyclin B1 MO injected oocytes (n=26) demonstrating that absolute CDK1 activity does not differ until approximately 2-2.5 hours ahead of polar body extrusion. Time 0 = PB1 extrusion in control oocytes.

Destruction of D-box-B1-V in untreated (n=20) and cyclin B1 MO treated oocytes (n=31) as; **(B)** raw data traces aligned at GVBD, thin traces represent individual oocytes, while heavy traces represent the average; **(C)** GVBD aligned data normalised to 100% at the maximum fluorescence prior to PB1 extrusion. **(D)** Mean destruction profiles of securin-V in control oocytes (n=15 oocytes) and cyclin B1 MO oocytes (n=15 oocytes). Error bars ± SEM.

Figure S7. Related to Fig. 6. Spindle parameter measurements in control and cyclin B1 MO oocytes. Box plots of relative spindle length and width in control oocytes (green) and cyclin B1 MO oocytes (purple) at **(A)** 5.5hrs post GVBD and **(B)** 'pre-anaphase' (the last image collected before anaphase). **(C)** Box plots showing relative chromosome dispersion in control (green) and cyclin B1 MO oocytes (purple) at 5.5hrs post GVBD and 'pre-anaphase'. **(D)** Box plots showing the relative distance of the spindle from the cortex in control (green) and cyclin B1 MO oocytes (purple) at 5.5hrs post GVBD and 'pre-anaphase'. * denotes a significant difference in means P<0.05. ** denotes a significant difference in means P<0.005. **(E)** Given that cyclin B1 MO treated oocytes extrude PBs earlier than control oocytes (~40 minutes earlier), a pre-anaphase measurement (taken a maximum of 15 minutes ahead of anaphase) in cyclin B1 MO oocytes could be argued to be more closely analogous to images of control oocytes taken 30-45 minutes earlier (45-60 minutes' pre-anaphase). Therefore, we quantified changes in chromosome dispersion at earlier time points in control oocytes. We suggest that chromosomes do not simply condense on the metaphase plate within the final 30-45 minutes. Instead, as previously reported (Kitajima et al., 2011), this is a gradual process. We suggest that in cyclin B1 MO oocytes the lack of chromosome congression at anaphase is not simply due to premature cytokinesis.

Table S1. Related to Figs. 5 and 6. P-values summarising the significance of differences in measurements of spindle morphology between time points. Between 5.5 hours post GVBD and pre- anaphase, differences in spindle length and width were not judged to change in either control or cyclin B1 MO oocytes. Between the same time points, chromosomes congressed in control oocytes (P=0.003, green). However, a much more modest reduction in the dispersion of chromosomes was not judged to be significant in cyclin B1 MO oocytes (P=0.097, pink). In both control and cyclin B1 MO oocytes the spindle moved significantly closer to the cortex of the oocyte.

Figure S1

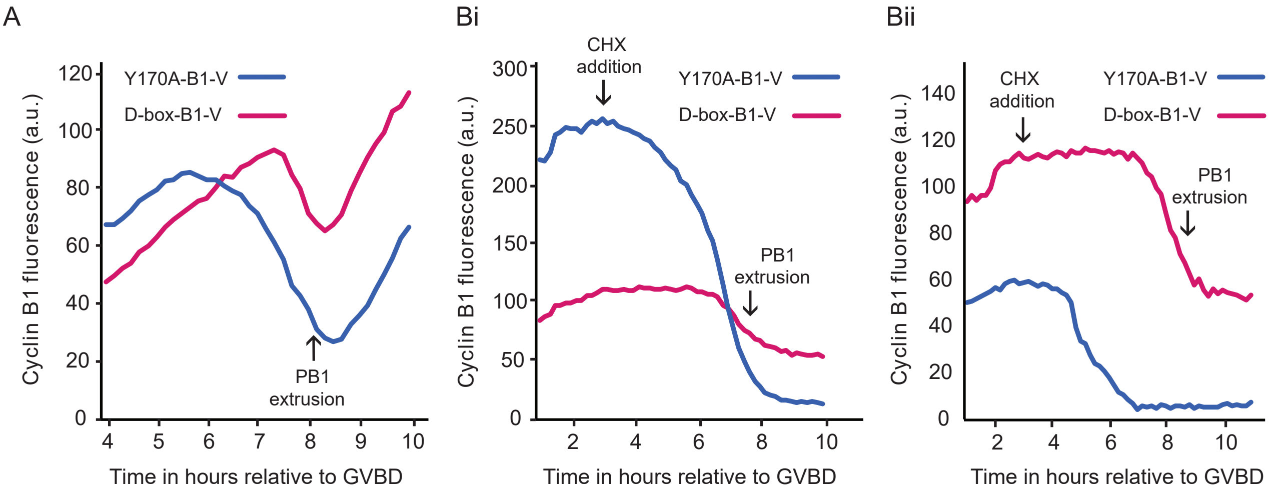
A
Bi
Bii
Y170A-B1-V
D-box-B1-V
CHX addition
PB1 extrusion
Cyclin B1 fluorescence (a.u.)
Time in hours relative to GVBD

Figure S2

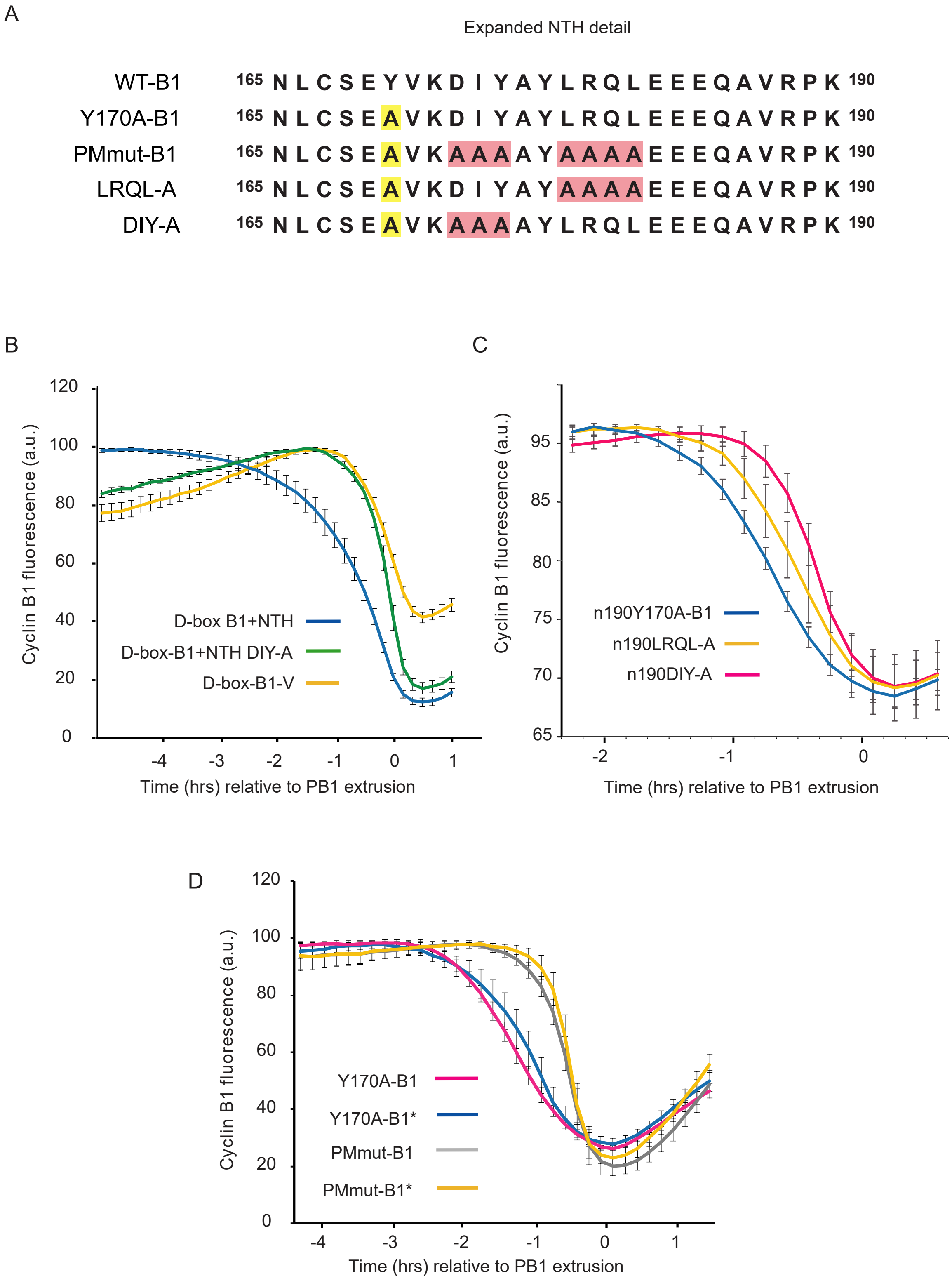
A
Expanded NTH detail
WT-B1 165 N L C S E Y V K D I Y A Y L R Q L E E E Q A V R P K 190
Y170A-B1 165 N L C S E A V K D I Y A Y L R Q L E E E Q A V R P K 190
PMmut-B1 165 N L C S E A V K A A A A Y A A A A E E E Q A V R P K 190
LRQL-A 165 N L C S E A V K D I Y A Y A A A A E E E Q A V R P K 190
DIY-A 165 N L C S E A V K A A A A Y L R Q L E E E Q A V R P K 190
B
Cyclin B1 fluorescence (a.u.)
Time (hrs) relative to PB1 extrusion
D-box B1+NTH
D-box-B1+NTH DIY-A
D-box-B1-V
C
Cyclin B1 fluorescence (a.u.)
Time (hrs) relative to PB1 extrusion
n190Y170A-B1
n190LRQL-A
n190DIY-A
D
Cyclin B1 fluorescence (a.u.)
Time (hrs) relative to PB1 extrusion
Y170A-B1
Y170A-B1*
PMmut-B1
PMmut-B1*

Figure S3

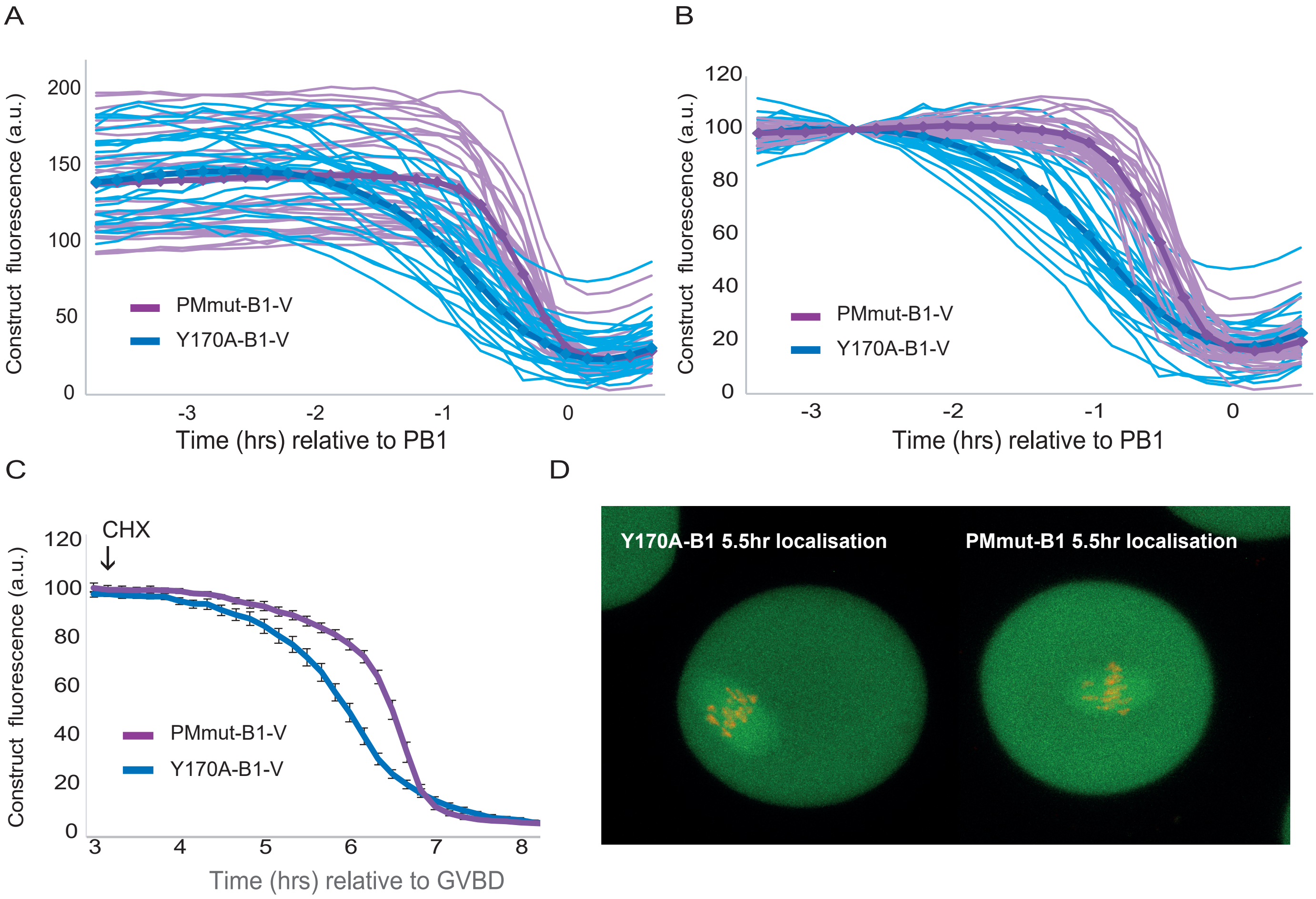
A
Construct fluorescence (a.u.)
200
150
100
50
0
PMmut-B1-V
Y170A-B1-V
-3
-2
-1
0
Time (hrs) relative to PB1
B
Construct fluorescence (a.u.)
120
100
80
60
40
20
0
PMmut-B1-V
Y170A-B1-V
-3
-2
-1
0
Time (hrs) relative to PB1
C
CHX
Construct fluorescence (a.u.)
120
100
80
60
40
20
0
PMmut-B1-V
Y170A-B1-V
3
4
5
6
7
8
Time (hrs) relative to GVBD
D
Y170A-B1 5.5hr localisation
PMmut-B1 5.5hr localisation

Figure S4

A

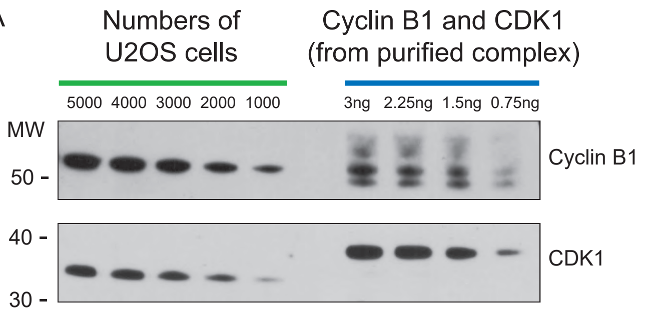

D

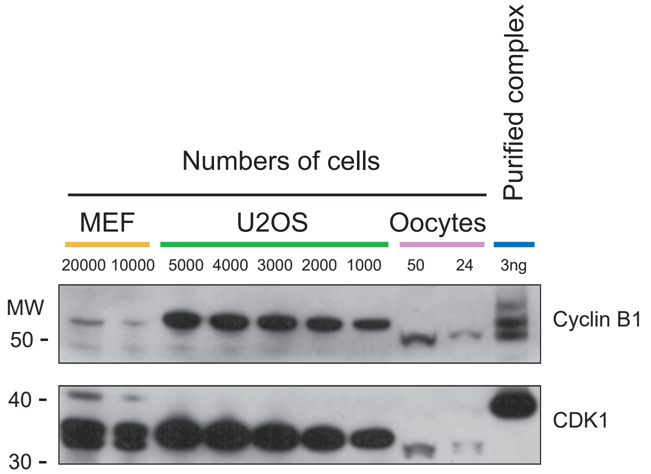

B

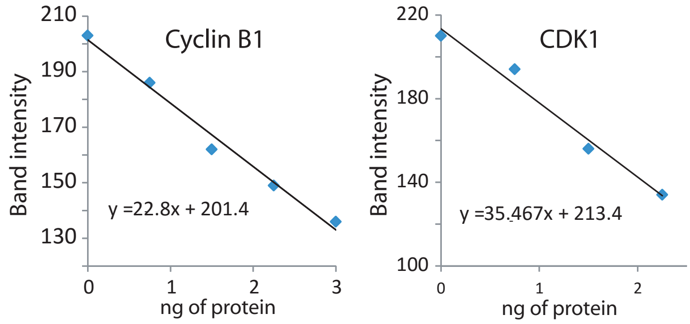

E

		Cyclin B1	CDK1
50 oocytes ~		815 U2OS cells	135 U2OS cells
1 oocyte ~		16.3 U2OS cells	2.7 U2OS cells
ng per single oocyte		11	1
nM		716	123
Ratio		6	1

C

U2OS cells	CDK1 band intensity	CDK1 in 1000 cells	Cyclin B1 band intensity	Cyclin B1 in 1000 cells
1000	203	*	185	0.72
2000	183	0.42	171	0.67
3000	171	0.40	156	0.66
4000	158	0.39	147	0.66
Average per 1000 cells		= 0.40 ng		= 0.68 ng

F

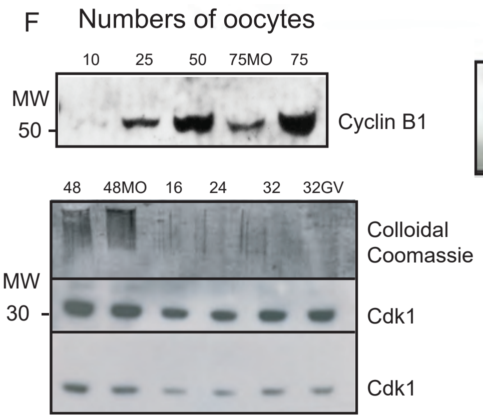

G

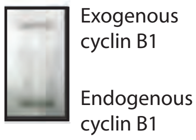

Figure S5

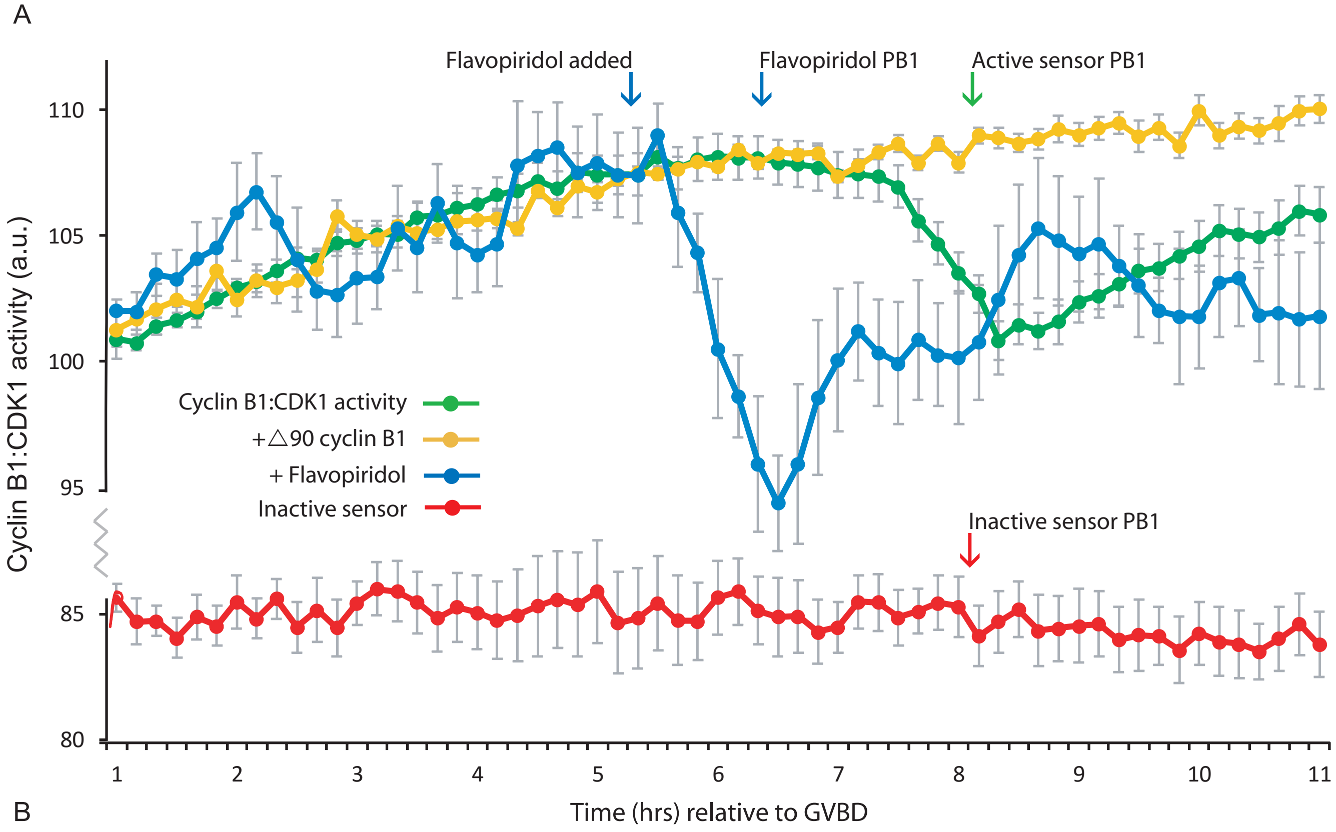
A
Flavopiridol added
Flavopiridol PB1
Active sensor PB1
Inactive sensor PB1
Cyclin B1:CDK1 activity (a.u.)
110
105
100
95
85
80
1
2
3
4
5
6
7
8
9
10
11
Time (hrs) relative to GVBD
Cyclin B1:CDK1 activity
+△90 cyclin B1
+ Flavopiridol
Inactive sensor

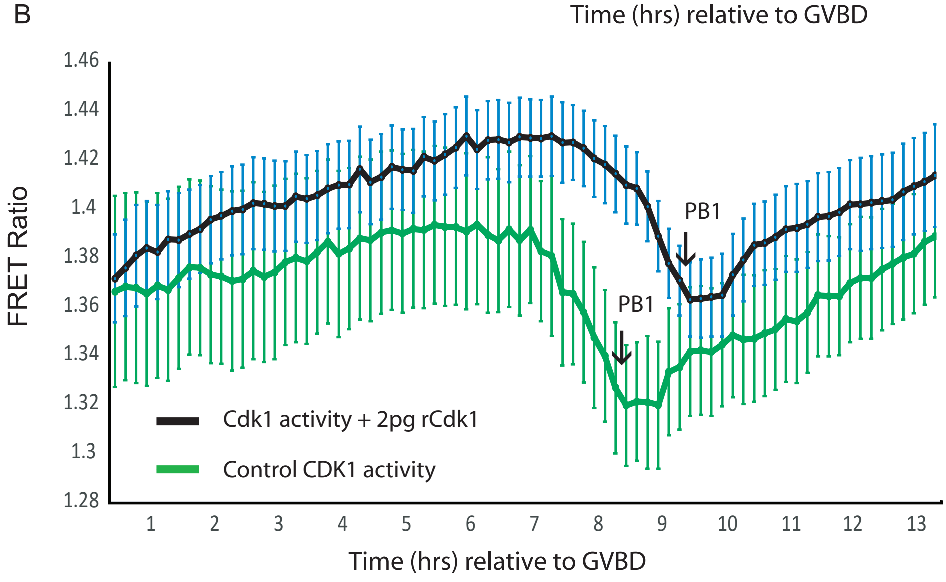
B
FRET Ratio
1.46
1.44
1.42
1.4
1.38
1.36
1.34
1.32
1.3
1.28
PB1
PB1
Cdk1 activity + 2pg rCdk1
Control CDK1 activity
1
2
3
4
5
6
7
8
9
10
11
12
13
Time (hrs) relative to GVBD

Figure S6

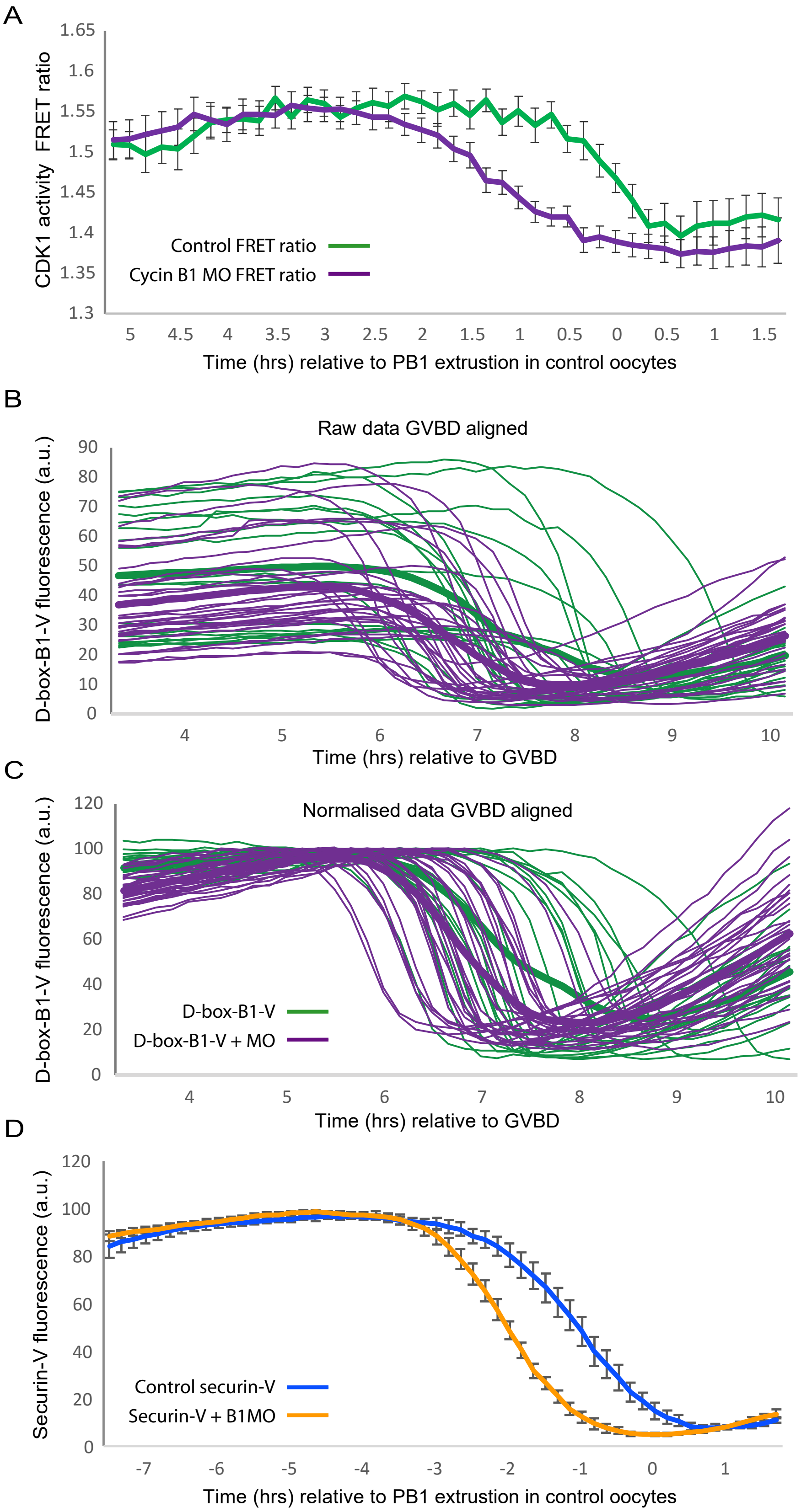
A
CDK1 activity FRET ratio
1.65
1.6
1.55
1.5
1.45
1.4
1.35
1.3
Control FRET ratio
Cycin B1 MO FRET ratio
5 4.5 4 3.5 3 2.5 2 1.5 1 0.5 0 0.5 1 1.5
Time (hrs) relative to PB1 extrustion in control oocytes
B
Raw data GVBD aligned
D-box-B1-V fluorescence (a.u.)
90 80 70 60 50 40 30 20 10 0
4 5 6 7 8 9 10
Time (hrs) relative to GVBD
C
Normalised data GVBD aligned
D-box-B1-V fluorescence (a.u.)
120 100 80 60 40 20 0
D-box-B1-V
D-box-B1-V + MO
4 5 6 7 8 9 10
Time (hrs) relative to GVBD
D
Securin-V fluorescence (a.u.)
120 100 80 60 40 20 0
Control securin-V
Securin-V + B1MO
-7 -6 -5 -4 -3 -2 -1 0 1
Time (hrs) relative to PB1 extrustion in control oocytes

Figure S7

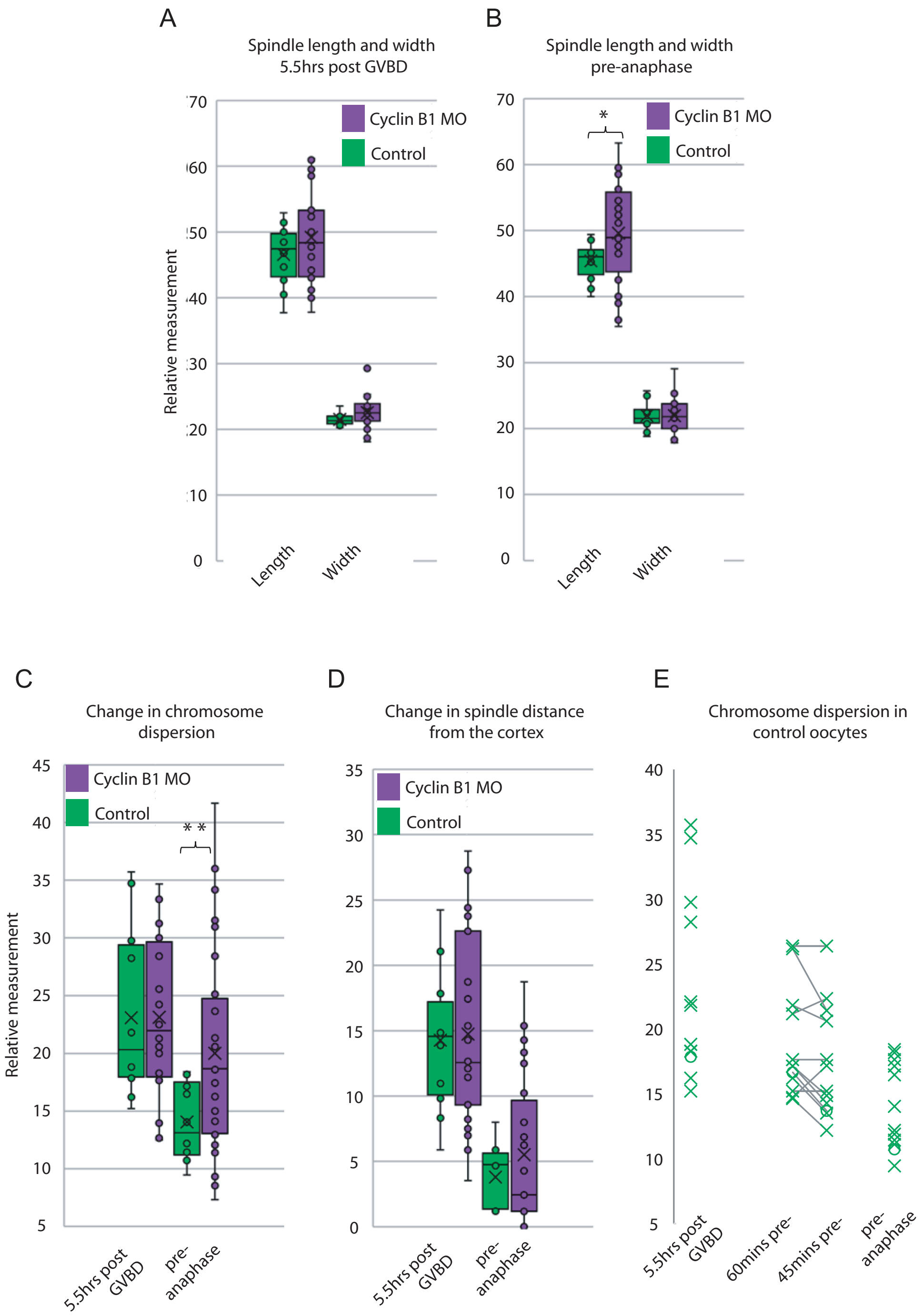
A
Spindle length and width
5.5hrs post GVBD
Relative measurement
Cyclin B1 MO
Control
Length
Width
B
Spindle length and width
pre-anaphase
Cyclin B1 MO
Control
Length
Width
C
Change in chromosome dispersion
Relative measurement
Cyclin B1 MO
Control
5.5hrs post GVBD
pre-anaphase
D
Change in spindle distance from the cortex
Cyclin B1 MO
Control
5.5hrs post GVBD
pre-anaphase
E
Chromosome dispersion in control oocytes
5.5hrs post GVBD
60mins pre-
45mins pre-
pre-anaphase

Table S1

**Paired data comparisons between 5.5 hrs post GVBD and pre-anaphase; equality of means**			
Parameter	*Treatment*	*P value*	*Test*
Spindle length	Control	0.134	t-test (2-tailed)
Spindle length	Cyclin B1 MO	0.759	t-test (2-tailed)
Spindle width	Control	0.527	t-test (2-tailed)
Spindle width	Cyclin B1 MO	0.520	t-test (2-tailed)
Chromosome dispersion	Control	0.003	t-test (2-tailed)
Chromosome dispersion	Cyclin B1 MO	0.097	t-test (2-tailed)
Spindle distance from cortex	Control	0.002	Wilcoxon Signed Rank
Spindle distance from cortex	Cyclin B1 MO	0.000	Wilcoxon Signed Rank
